# Thermotolerance, virulence, and drug resistance of human pathogenic *Candida* species colonising plastic pollution in aquatic ecosystems

**DOI:** 10.1007/s11356-025-36558-2

**Published:** 2025-06-04

**Authors:** Rebecca Metcalf, Ayorinde Akinbobola, Luke Woodford, Richard S. Quilliam

**Affiliations:** Biological and Environmental Sciences, Faculty of Natural Sciences, University of Stirling, Stirling, FK9 4LA UK

**Keywords:** Antifungal resistance, Environmental pollution, Fungal pathogens, Plastisphere, Pathogenic yeast

## Abstract

Recently, six species of the pathogenic yeast *Candida* have been classified as priority fungal pathogens by the World Health Organisation (WHO), yet the persistence and niches of pathogenic species of *Candida* in the environment remains unclear. And despite the ability of *Candida* to form strong biofilms on plastics in clinical settings, the potential for plastics in the environment to facilitate survival and dissemination of *Candida* as has not been determined. Here, we have collected plastic pollutants from freshwater, estuarine, and marine environments and successfully isolated five pathogenic species of *Candida*, including three that are on the WHO Fungal Priority Pathogens list (*C. glabrata, C. tropicalis, Pichia kudriavzevii* (formerly known as *Candida krusei*). All environmental isolates were resistant to at least one antifungal drug, thermotolerant to human body temperature, and showed pathogenicity in a *Galleria mellonella* model of infection. In addition, polyethylene (PE) plastic pellets were deployed upstream and downstream of a wastewater discharge pipe in a small freshwater stream. Although all pellets became colonised by pathogenic species of *Candida*, there were clear differences in species prevalence upstream and downstream of the effluent, indicating a clear point source for *Candida* discharge into surface waters. The global incidence of candidiasis, particularly by drug resistant strains, is increasing, and it is therefore critical that we increase our focus on the environmental reservoirs of these pathogens and continue to improve our understanding of their environmental persistence and subsequent transfer routes to humans.

**Supplementary Information:**

The online version contains supplementary material available at 10.1007/s11356-025-36558-2.

## Introduction

Plastic pollution in the environment is of major global concern, with an estimated 390 million metric tonnes of plastic being produced annually, and production predicted to double in the next 20 years (Plastics Europe 2022). Only a small proportion of the total plastic produced globally is recycled, which results in large quantities of plastic waste ending up in the environment (Geyer et al. 2017; Kibria et al. 2023). Plastics are inherently recalcitrant to degradation and, as such, plastic pollutants can negatively impact both terrestrial and aquatic ecosystems (Li et al. 2016; Kibria et al. 2023). Importantly, once in the environment, plastic surfaces become rapidly colonised by microbial biofilm comprised of complex microbial communities (Zettler et al. 2013). Such ‘plastisphere’ communities can contain (or even enrich) human pathogens, which can then be disseminated within different environmental matrices (Junaid et al. 2022). The majority of plastisphere research has focussed on prokaryotic communities, including human bacterial pathogens (Jiang et al. 2018); however, eukaryotic microorganisms, including human fungal pathogens can also associate with the plastisphere (Gkoutselis et al. 2021).

Millions of severe infections and deaths are caused annually by fungal pathogens (Denning 2024; Kainz et al. 2020). One of the most important fungal infections is invasive candidiasis, caused by several species of the yeast *Candida*, with an estimated 700,000 cases annually (Bongomin et al. 2017). Candidiasis also includes less severe cutaneous and mucosal infections, such as thrush. Globally, infections due to potentially pathogenic *Candida* species are increasing, due in part to the emergence of more virulent strains of *Candida* (Siscar-Lewin et al. 2022), which is further compounded by the simultaneous emergence of pathogenic strains of drug-resistant *Candida* (Fisher et al. 2022; Parslow and Thornton 2022). This has led the WHO to add six species of *Candida* to the Fungal Pathogen Priority list (WHO, 2022), i.e., *Candida* (*Candidozyma*) *auris* and *C. albicans* both classed as Critical priority; *C. glabrata* (*Nakaseomyces glabrata*), *C. parapsilosis,* and *C. tropicalis,* all classed as High priority; and *C. krusei* (*Pichia kudriavzevii*) classed as Medium priority.

Understanding the source of infection and interaction of fungal pathogens within the environment is important for the effective management of fungal infections (Fisher et al. 2024), particularly as *Candida* is notorious for forming strong biofilms on plastic surfaces in healthcare settings (Estivill et al. 2011). Most of the approximately 200 species of *Candida* are non-pathogenic and persist in the environment, e.g., in water and soil, or associated with food, plants or insects (Lachance et al. 2011). However, several species are opportunistically pathogenic, taking advantage of the opportunity to colonise humans and cause disease (Ekdahl et al. 2023). *Candida* can survive the transition through wastewater treatment plants (WWTPs) (Assress et al. 2019) and persist in various environmental matrices (e.g., coastal wetlands (*C. auris*), freshwater (*C. glabrata, P. kudriavzevii, C. tropicalis*), seawater (*C. albicans, C. tropicalis*), and soil (*C. albicans, C. glabrata*; *C. tropicalis*) (Akinbobola et al. 2023; Arora et al. 2021; Brandao et al. 2010; Chen et al. 2009; Medeiros et al. 2008; Fotedar et al. 2022; Wójcik et al. 2013; Sautour et al. 2021). Therefore, during their transition through WWTPs and the environment, pathogenic species of *Candida* could encounter and colonise environmental plastic pollution.

Like other yeast species, *Candida* spp. are known to possess several features and mechanisms (e.g., epithelial adhesin expression, hydrolytic enzyme secretion) which enable them to successfully adhere and form biofilms (Silva et al. 2011). *C. parapsilosis* readily forms biofilm on plastics in clinical settings (Gómez-Molero et al., 2021), and previous studies with *C. auris* and *C. parapsilosis* have demonstrated that these species can also adhere to plastic surfaces under environmental mesocosm conditions (Oliveira et al. 2022; Dire et al. 2023; Akinbobola et al. 2024); whilst ITS sequencing has shown that *Candida* spp. are present in the plastisphere (Baker et al. 2024; Wallbank et al. 2022). Therefore, plastic pollutants could constitute an important vehicle for the dissemination of human pathogenic *Candida* in the environment with significant implications for the epidemiology and environmental management of *Candida* infections. In order to determine the co-pollutant risk of plastic pollutants to human health, we have collected and screened different types of plastic pollution in marine, estuarine, and freshwater environments for pathogenic species of *Candida*, and assessed these isolates for their thermotolerance, anti-fungal drug resistance, and their pathogenicity in a *Galleria* model of infection. Additionally, we have investigated the potential for wastewater discharge to act as a point source for the introduction of pathogenic *Candida* into surface waters by deploying cages containing polyethylene (PE) plastic pellets upstream and downstream of an effluent pipe discharging from a WWTP, and screening for subsequent colonisation by pathogenic species of *Candida*.

## Materials and methods

### Sample collection and processing

**Fig. 1:**
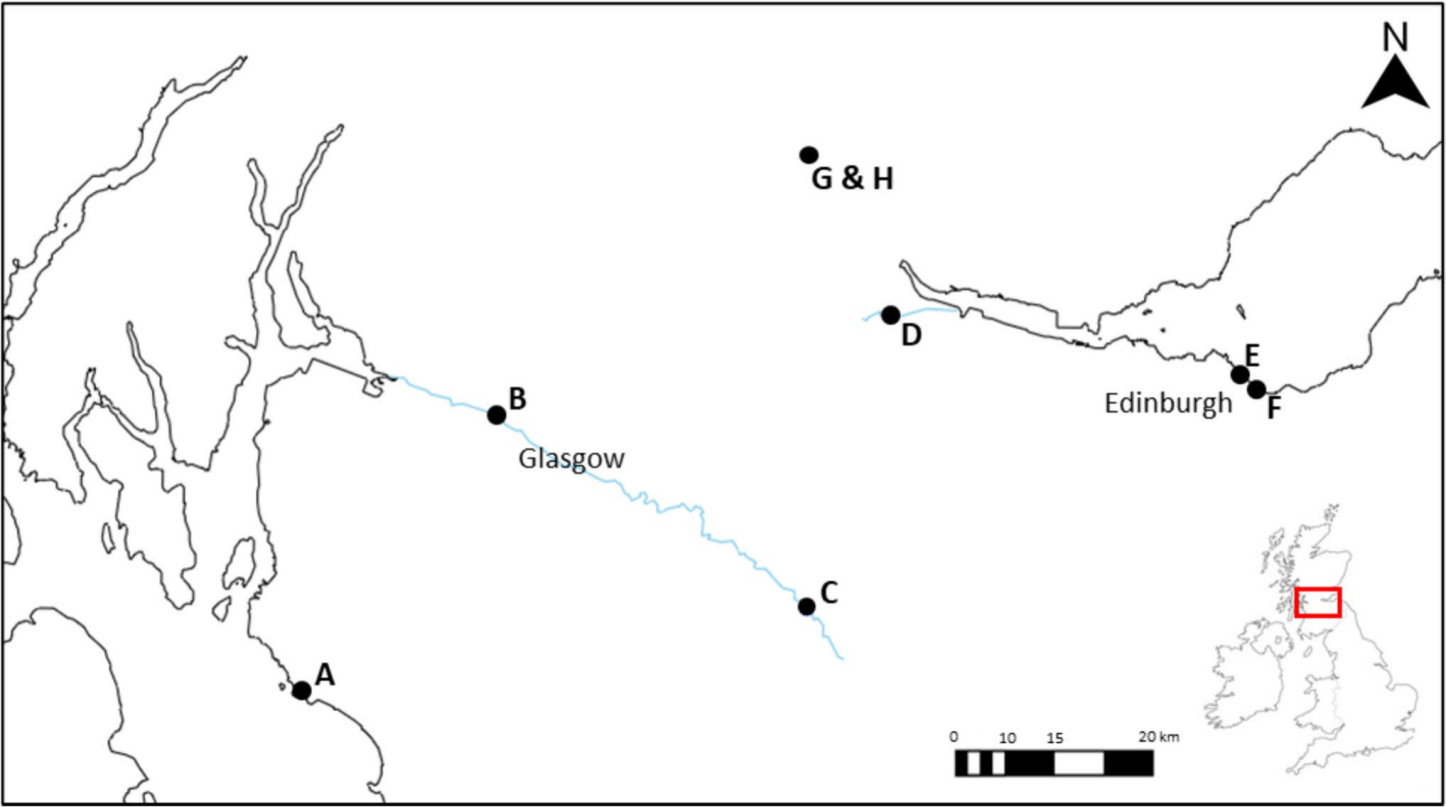
Sampling sites (black circles) in Central Scotland, UK. Additional site details are given in Table S1

Plastic debris was collected from six sites (A–F) on three separate days (March–May 2023; Fig. 1; Table S1). The sites included marine, estuarine, and freshwater beaches on the east (Firth) and west (Clyde) catchments in central Scotland, UK. Two of the beaches sampled were designated bathing water beaches, regulated under the EU Bathing Water Directive (BWD) 2006/7/EC. Central Scotland contains several large urban centres (e.g., Glasgow, Edinburgh) and has the highest population density in Scotland (population 4.2 million). At each site, items of plastic pollution were collected using sterile forceps and placed into sterile Ziplock bags. All samples were stored at 4 °C and processed within 24 h. Plastic samples were sorted into composite samples of either ‘hard plastics’ (High density polyethylene [HDPE] and Polyethylene terephthalate [PET]) such as cotton bud sticks, tampon applicators, and plastic fragments; ‘soft plastics’ (Low-density polyethylene [LDPE], Polypropylene [PP] and Polyethylene (PE) such as plastic bags, food wrappers, wet wipes); or ‘Polystyrene’ (PS).

### Point source discharge of *Candida* in receiving waters

To determine the role of wastewater discharge for introducing pathogenic *Candida* species into the environment, 4-mm PE beads (Goodfellow, UK) were placed in sterile stainless-steel cages in large metal baskets upstream (site G) and downstream (site H) of a waste-water treatment plant (WWTP) effluent pipe (Table S1) in May 2024. PE was chosen for this experiment as it is the most commonly produced plastic polymer globally (Erni-Cassola et al. 2019). The metal baskets were suspended in the river using fishing line approximately 15 m upstream of, and directly next to the WWTP effluent pipe. Plastic pellets were recovered from the baskets at days 1, 4, and 8, placed in sterile Ziplock bags, and processed on the same day as collection.

### Recovery of *Candida spp.* using selective media

Different quantities of plastic types were collected from sites A–F. Replicate composite samples of each plastic type (13 hard plastics (e.g., PET); 13 soft plastics (e.g., PE); 1 polystyrene; 1 wet wipe; 1 cotton bud stick) from each site were pre-enriched in 100-ml yeast extract peptone dextrose (YPD) broth supplemented with antibiotics (gentamicin 50 mg/L, chloramphenicol 50 mg/L; Sigma-Aldrich, USA) and incubated at 30 °C for 48 h in a shaking incubator (120 rpm; Incu-Shake MIDI Benchtop Shaking Incubator, SciQuip, UK). After incubation, 100 µl of the overnight culture and a 1:1000 diluent of the same culture was separately spread onto Sabouraud glucose agar with 50 mg/L chloramphenicol (SGA; Sigma-Aldrich, USA) supplemented with gentamicin 50 mg/L, and fluconazole at three different concentrations (0, 16, 64 mg/L) to select for fluconazole sensitive, moderately resistant and resistant strains. Plates were inverted and incubated for 48 h (30 °C). Colonies were then selected based on different morphologies on Sabouraud glucose agar and streaked onto CHROMagar™ *Candida* Plus agar (CHROMagar™, France) and incubated (30 °C, 48 h). *Candida* species were presumptively identified based on colony colour and morphology on CHROMagar™ *Candida* Plus agar. Twenty-seven colonies were isolated, and glycerol stocks (final concentration 40% glycerol) prepared and frozen at − 20 °C.

Cages collected from sites G and H were washed in 100 ml MilliQ water (Millipore) by rinsing three times. Fifty pieces of plastic (four independent replicates) were removed from each cage and placed in a sterile 30-ml glass universal with 5 ml of pre-enrichment broth of YPD, supplemented with 50 mg/L of chloramphenicol and gentamycin (Sigma-Aldrich, USA), and incubated on a shaker at 37 °C for 48 h. Following incubation, serial dilutions of each broth were performed using sterile PBS, then 100 µl of 10^−3^ and 10^−5^ dilutions were spread on CHROMagar *Candida* Plus agar (CHROMagar™, France). Plates were incubated (37 °C for 48 h) and single colonies showing morphological characteristics of *Candida* species were isolated for PCR screening, sanger sequencing and minimum inhibitory concentration testing. The single colonies were picked and mixed in 1:1 YPD and 80% glycerol and stored at − 80 °C.

## Characterisation of *Candida* isolates from environmental plastic pollution

### PCR and sequencing for *Candida* identification

Glycerol stocks of 27 selected isolates were grown overnight in 5-ml YPD (120 rpm, 37 °C, 24 h). Colony PCR was carried out with primers from Carvalho et al. (2007) targeting the ITS region to identify the five common pathogenic species of *Candida*, i.e., *C. albicans*, *C. glabrata*, *P. kudriavzevii*, *C. parapsilosis*, and *C. tropicalis*. Amplification reactions consisted of 12.5-µL multiplex master mix (New England Biolabs, UK), 1 µL each of the forward and reverse primers (10 µmol/L), 2 µL of each DNA sample and 8.5 µL of sterile water in a final reaction volume of 25 µL. PCR amplification was carried out in a thermal cycler (Veriti 96-well thermal cycler, Applied Biosystems, USA) using the following cycle: 10-min initial denaturation at 94 °C, followed by 40 cycles of 94 °C for 15 s, 55 °C for 30 s and 65 °C for 45 s, with a final extension of 65 °C for 10 min. All PCR products were run down a 2% agarose gel using GelRedÒ staining (Biotium, USA) and visualised under UV. Different amplicon sizes were used to differentiate between distinct *Candida* species: *C. albicans* (446 bp), *C. glabrata* (839 bp), *P. kudriavzevii* (169 bp), *C. parapsilosis* (370 bp) and *C. tropicalis* (507 bp).

To further confirm the identity of each isolate, the ITS1 region was amplified and sequenced. Additionally, a selection of suspected *Candida* colonies from Sites G and H were selected for screening. Firstly, DNA was extracted using DNeasy Tissue kit (Qiagen, Germany) and eluted in 200 µL Buffer AE, and primers from Trost et al. (2004) used to amplify the ITS1 region (Forward primer: 5′-GTCAAACTTGGTCATTTA-3′; Reverse primer: 5′-TTCTTTTCCTCCGCTTATTG-3′). Amplification reactions consisted of 2 × *taq* PCR master mix (Qiagen, Germany), 0.4-µM primer and 5 µL of each DNA sample in a final reaction volume of 25 µL. PCR amplification comprised an initial denaturation step at 94 °C for 3 min followed by 34 cycles of amplification (94 °C for 30 s, 50 °C for 30 s and 72 °C for 1 min) followed by a final extension at 72 °C for 10 min. PCR products were purified using a QIAquick PCR Purification kit (Qiagen, Germany), and eluted in 50 µL of elution buffer. All purified PCR products underwent Sanger sequencing using Applied Biosystems 3730 DNA analysers (DNA Sequencing and Services, Dundee, UK). Species ID was confirmed using NCBI’s Basic Local Alignment Search Tool (BLAST; NCBI, USA).

### Pathogenicity

To determine virulence, each isolate was introduced into a *Galleria mellonella* model of infection (Romera et al. 2020). Healthy larvae of *G. mellonella* (Greater wax moth; Livefood, UK) between 2.0 and 2.5 cm in length were selected, kept in darkness at 15 °C and used within one week of purchase. Glycerol stocks of each *Candida* spp. isolate were cultured on SGA agar (37 °C, 24 h), and distinct colonies selected and grown in 5-ml YPD (37 °C, 24 h, 120 rpm). To ensure that cells were in their exponential growth phase when injected into *G. mellonella*, 1-ml overnight cultures were added to 5-ml YPD and grown to an OD_570_ of 0.7. Cells were then centrifuged (4000 rpm, 4 min) before being washed and resuspended in PBS. Replicate groups of 10 larvae were injected with 10 µL of *Candida* cells (10^5^ CFU/larvae) into the hemocoel via the last right pro-limb using a sterile 100-µL Hamilton syringe (Bonaduz, Switzerland) with a 0.6 × 30 mm needle. All experiments were conducted in biological triplicate. Needles were flushed with ethanol followed by PBS to sterilise them between samples. An inoculation of 10-µL PBS was used as a control to account for mortality caused by physical injury or infection by contamination. Following injection, larvae were incubated at 37 °C, and survival evaluated every 24 h for a total of 120 h. Larvae were considered dead when they did not respond to a touch stimulus.

### Thermotolerance

To determine the thermotolerance profile, each isolate underwent an initial incubation at 18 °C (simulating an environmental temperature) or 38 °C (simulating an extreme of human body temperature) for 24 h, before being transferred to one of three different temperatures (18, 28 or 38 °C) where their growth was measured. Colonies were selected from SGA plates and grown overnight in 5-ml YPD (37 °C, 24 h, 120 rpm), and cells centrifuged (4000 rpm, 8 min), washed and resuspended in phosphate buffered saline (PBS). Cell concentrations were adjusted by dilution in sterile distilled water to give a final concentration of approximately 10^5^ CFU/ml (PBS serial dilutions were plated on SGA agar and incubated for retrospective enumeration). Cells (20 ml) of each isolate were added to 180 ml YPD broth in 96-well plates (*n* = 3), and plates incubated at either 18, 28 or 38 °C for 24 h. I-Button temperature logger chips (iButtonLink, WI, 176 USA) were placed into each incubator to monitor the temperature throughout incubation. Absorbance at 570 nm was measured before and after incubation in a spectrophotometer (Infinite M200 plate reader; Tecan, Switzerland) to determine growth of the isolates.

### Antifungal drug resistance

Each *Candida* isolate, including those isolated from site G and H, was subjected to minimum inhibitory concentration (MIC) analysis to determine antifungal resistance following the European Committee on Antimicrobial susceptibility testing (EUCAST) antifungal MIC method for yeasts (Arendrup et al. 2020). Resistance to four antifungals at ten concentrations was examined: amphotericin B (0.008–4 mg/L), caspofungin (0.008–4 mg/L), fluconazole (0.125–64 mg/L), voriconazole (0.008–4 mg/L) (Thermoscientific, USA). Briefly, 96-well plates were filled with 100-µL double strength RPMI 1640 (2% glucose, Sigma-Aldrich, USA) containing the different concentrations of antifungal drugs. Distinct colonies were selected from SGA plates and grown overnight in 5 ml YPD (37 °C, 24 h, 120 rpm). Cells were recovered by centrifugation (4000 rpm, 8 min), washed and resuspended in PBS, and the concentration adjusted to approximately 10^5^ cells/ml. Adjusted cultures (100 µL per well) of each isolate were added to 96-well plates and incubated without agitation at 35 °C for 24 h. Control wells contained sterile drug-free medium, with 100 µL of the same adjusted cultures. Absorbance at 530 nm was measured in a spectrophotometer (Infinite M200 plate reader; Tecan, Switzerland) to determine growth of the isolates. The MIC of amphotericin B was determined as the lowest concentration giving rise to an inhibition of 90% of growth compared to the drug-free control. The MIC of caspofungin, fluconazole and voriconazole was determined as the lowest concentration giving inhibition of 50% growth compared to the drug-free control.

### Statistical analysis

Statistical analysis was conducted in R Studio version 3.3.2 (R Core Team, 2021). Student’s *t*-tests and analysis of variance (ANOVA) were used to test for differences in pathogenicity, thermotolerance, and antifungal resistance. All data were tested for distribution and homogeneity of variance (Shapiro–Wilk and Levene’s) before parametric tests were used. Where assumptions were not met, data were either log transformed, or non-parametric Mann–Whitney *U* or Kruskal–Wallis tests used. Tukey’s and Fisher’s LSD post hoc tests were used to compare groups. Data is reported as mean ± SE, and *P* values ≤ 0.05 are considered significant.

## Results

### Recovery of *Candida* from environmental plastic pollution

Five species of *Candida* (*C. glabrata*, *C. pseudolambica*, *C. sojae*, *C. tropicalis* and *P. kudriavzevii*) were isolated from the surfaces of plastic pollutants at sites A-F (Table 1), with three of these species (*C. glabrata*, *C. tropicalis* and *P. kudriavzevii*) listed on the WHO ‘Fungal Priority Pathogens’ list (WHO, 2022). Twenty isolates of *C. glabrata* were isolated from seven different sites, which included all three environments, i.e., marine, estuarine, and freshwater. In contrast, most (7/11) *C. tropicalis* isolates were detected on plastics at one of the marine sites. *P. kudriavzevii* was only detected on plastic in freshwater and estuarine sites in the Clyde catchment; both these isolates showed the most similarity to the same accession number (OW988737) despite being isolated from two geographically different sites. *Candida* species were also isolated from the plastic particles deployed at site G, upstream of the effluent pipe (*C. albicans* and *C. glabrata*) and site H, downstream of the effluent pipe, (*C. albicans*, *C. glabrata*, *C. tropicalis* and *P. kudriavzevii*).

**Table 1:** *Candida* isolates from environmental plastic pollution. Species determined by top Genbank accession match and phylogenetic analysis (Fig S2).

Isolate number	Site	Site type	Species	Top GenBankAccession match	Plastic type	GenBankAccession Number
1	A	Marine	*C. glabrata*	OW988792	Soft plastic	PQ159241
2	B	Estuarine	*C. glabrata*	ON016558	Soft plastic	PQ159242
3	B	Estuarine	*C. glabrata*	ON391970	Polystyrene	PQ159243
4	B	Estuarine	*C. glabrata*	LC317501	Hard plastic	PQ159244
5	B	Estuarine	*C. glabrata*	MG560156	Hard plastic	PQ159245
6	B	Estuarine	*C. glabrata*	KX008750	Soft plastic	PQ159246
7	B	Estuarine	*P. kudriavzevii*	OW988737	Hard plastic	PQ159257
8	B	Estuarine	*C. sojae*	NR_137087	Hard plastic	PQ159260
9	C	Freshwater	*C. glabrata*	OP876825	Soft plastic	PQ159247
10	C	Freshwater	*C. glabrata*	OP850582	Soft plastic	PQ159248
11	C	Freshwater	*C. glabrata*	OP850582	Hard plastic	PQ159249
12	C	Freshwater	*C. glabrata*	OP850582	Hard plastic	PQ159250
13	C	Freshwater	*C. glabrata*	NR_130691	Soft plastic	PQ159251
14	C	Freshwater	*C. glabrata*	KU987871	Soft plastic	PQ159252
15	C	Freshwater	*C. glabrata*	MF033154	Hard plastic	PQ159253
16	C	Freshwater	*P. kudriavzevii*	OW988737	Soft plastic	PQ159258
17	C	Freshwater	*C. pseudolambica*	MW895903	Soft plastic	PQ159259
18	D	Freshwater	*C. glabrata*	MF187236	Soft plastic	PQ159254
19	E	Marine	*C. glabrata*	MZ255116	Soft plastic	PQ159255
20	E	Marine	*C. glabrata*	OW988778	Soft plastic	PQ159256
21	E	Marine	*C. tropicalis*	MK748468	Wet wipes	PQ159261
22	E	Marine	*C. tropicalis*	JKY102470	Wet wipes	PQ159262
23	E	Marine	*C. tropicalis*	OP627182	Hard plastic	PQ159263
24	E	Marine	*C. tropicalis*	MZ648456	Hard plastic	PQ159264
25	E	Marine	*C. tropicalis*	MH628218	Hard plastic	PQ159265
26	F	Marine	*C. tropicalis*	OW986301	Hard plastic	PQ159266
27	F	Marine	*C. tropicalis*	OW986301	Hard plastic	PQ159267
28	G	Upstream of WWTP	*C. albicans*	LC601970.1	Polyethylene	PQ651727
29	G	Upstream of WWTP	*C. albicans*	LC537155.1	Polyethylene	PQ651728
30	G	Upstream of WWTP	*C. glabrata*	GQ376081.1	Polyethylene	PQ651732
31	G	Upstream of WWTP	*C. albicans*	PP352569.1	Polyethylene	PQ651724
32	H	Downstream of WWTP	*C. albicans*	OP025165.1	Polyethylene	PQ651731
33	H	Downstream of WWTP	*C. tropicalis*	PP808755.1	Polyethylene	PQ651721
34	H	Downstream of WWTP	*C. tropicalis*	OR018819.1	Polyethylene	PQ651725
35	H	Downstream of WWTP	*C. tropicalis*	FJ697166.1	Polyethylene	PQ651726
36	H	Downstream of WWTP	*C. tropicalis*	PP808743.1	Polyethylene	PQ651733
37	H	Downstream of WWTP	*P. kudriavzevii*	MH979676.1	Polyethylene	PQ651722
38	H	Downstream of WWTP	*C. glabrata*	LR757911.1	Polyethylene	PQ651723
39	H	Downstream of WWTP	*C. glabrata*	ON016561.1	Polyethylene	PQ651730
40	H	Downstream of WWTP	*C. glabrata*	LC317498.1	Polyethylene	PQ651729

‘Hard plastics’, HDPE and PET; ‘soft plastics’ LDPE, PE and PP

### Pathogenicity

**Fig. 2:**
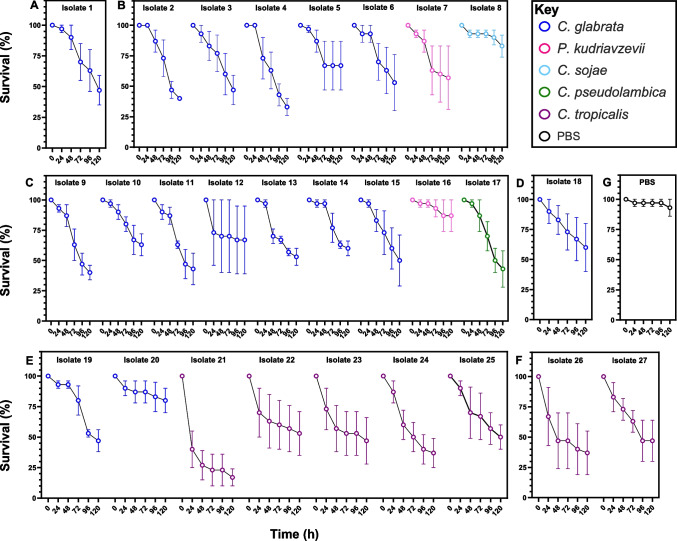
Virulence of *Candida* isolates colonising environmental plastic pollution (from Sites** A**–**F**) in a *Galleria mellonella* infection model. PBS controls are also included (**G**). Samples are coloured by *Candida* species with the isolate ID shown above. Data points (*n* = ten *G. mellonella* larvae) represent the mean of three independent biological replicates ± SE

All isolates were pathogenic in the *Galleria* larvae infection model, although this varied between the different isolates (Fig. 2). At 120 h after pathogen challenge, the percentage survival of *Galleria* larvae was significantly lower for all isolates compared to the PBS control (Fig. 2; Mann–Whitney *U*, W = 64, *p* < 0.01). The site where *Candida* had been isolated from had no significant influence on the percentage survival of challenged larvae at 120 h (ANOVA, F_5, 75_ = 0.562, *p* = 0.729); whilst species of *Candida* did have an effect on percentage survival at 120 h (ANOVA, F_4, 76_ = 3.223, *p* < 0.05), although the only significant difference was between *C. sojae* and *C. tropicalis* (Fisher’s LSD; difference in means = 42.4%).

### Thermotolerance

**Fig. 3:**
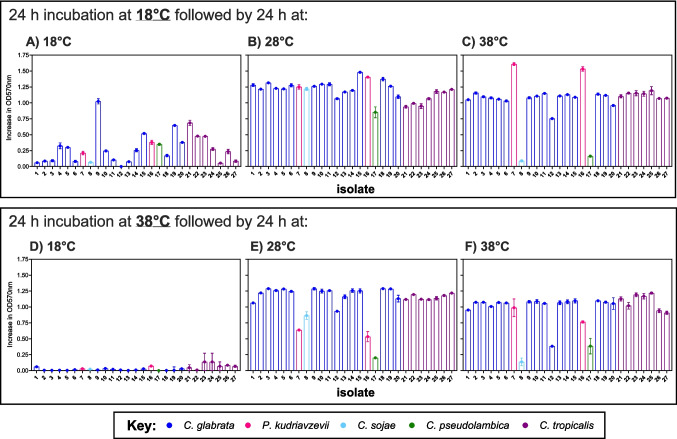
Thermotolerance of *Candida* isolated from environmental plastic pollution. Isolates were initially incubated for 24 h at 18 °C (**A**–**C**) or 38 °C (**D**–**F**), before subsequent transfer to 18 °C (**A**, **D**), 28 °C (**B**, **E**) and 38 °C (**C**, **F**) for a further 24 h. The different coloured bars represent the different species

Growth of environmental isolates of *Candida* was significantly affected by temperature (ANOVA, F_2, 177_ = 516.9, *p* < 0.001), with growth at 28 and 38 °C significantly higher than at 18 °C (Fig. 3). The isolates with the lowest levels of growth at 18 °C had initially been incubated at 38 °C (Fig. 3D), whereas previous acclimatisation at 18 °C produced higher rates of growth (Fig. 3A). In general, there was a significant effect on the rate of growth between *Candida* species (ANOVA, F_5, 174_ = 2.401, *p* < 0.05); however, the only significant difference in growth was between *C. glabrata* and *C. pseudolambica* (Fisher’s LSD; difference in means = 0.48). Two isolates (e.g., 17, *C. pseudolambica*; and 8, *C. sojae*) had low levels of growth at 38 °C (regardless of the pre-incubation temperature), suggesting that these species have a lower optimal growth temperature than the other *Candida* species, which are known to be opportunistic human pathogens. In contrast, growth of *P. kudriavzevii* (i.e., isolates 7 and 16) at 38 °C following an initial incubation at 18 °C had the highest levels of growth, suggesting that these environmental isolates could successfully acclimatise to the temperature of the human body. The nature of the sites where *Candida* species were isolated from had no significant influence on their growth profile or thermotolerance (ANOVA, F_6, 173_ = 0.109, *p* = 0.995).

### Antifungal resistance

**Fig. 4:**
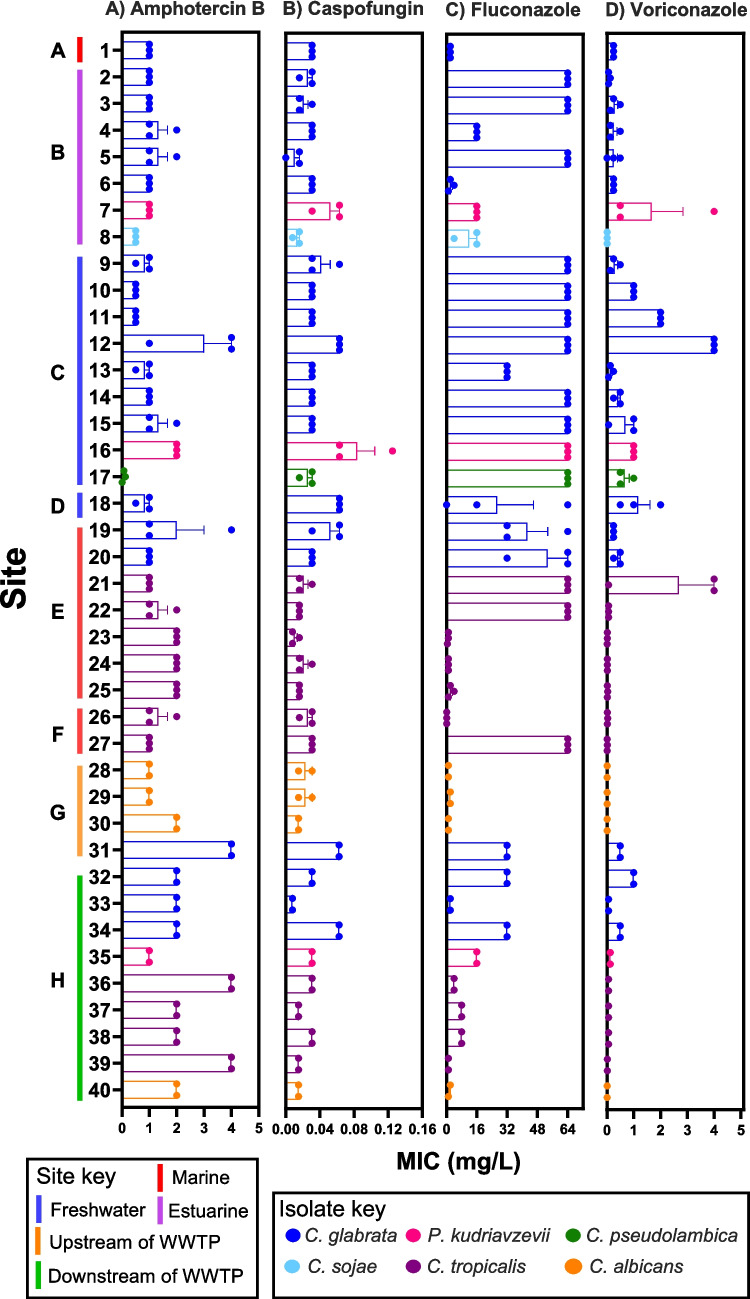
Minimum inhibitory concentration (MIC) of four antifungals against *Candida* isolates from environmental plastic pollution. (**A**) amphotericin B (0.008–4 mg/L); (**B**) caspofungin (0.008–4 mg/L); (**C**) fluconazole (0.125–64 mg/L); (**D**) voriconazole (0.008–4 mg/L). The different coloured bars represent the different *Candida* species. Isolates from sites G and H were only tested against fluconazole at 0.125–32 mg/L. There were three replicates per isolate, and error bars indicate the SE of the mean

All forty *Candida* isolates showed resistance to at least one of the four antifungals tested (amphotericin B, caspofungin, fluconazole, voriconazole), with a single isolate of *C. glabrata* from site C having high levels of resistance to all four antifungals (Fig. 4). However, isolates of *C. tropicalis* expressed a range of resistance profiles to fluconazole, i.e., three of the eleven isolates were resistant to concentrations of 64 mg/L, whilst the other eight isolates were only tolerant to concentrations between 0.8 and 8 mg/L (Fig. 4C). A similar pattern was observed for voriconazole, where ten of the eleven *C. tropicalis* isolates were highly susceptible (Fig. 4D). Two isolates of *C. tropicalis* (site E, samples 21 and 22) which showed high levels of resistance to fluconazole were both isolated from wet wipes, whilst the other strains of *C. tropicalis* from sites E and F were isolated from hard plastic (Table 1). The *C. albicans* isolates from site G (isolates 28–30) showed high susceptibility to fluconazole and voriconazole and typically had greater drug susceptibility than the other *Candida* strains isolated from the effluent pipe at site H. Species identity had an influence on the MIC (ANOVA, F_5,422_ = 2.882, *p* < 0.05); however, the only significant difference was between *C. glabrata* and *C. albicans* (*p* = 0.03). Overall, the location of site the *Candida* had been isolated from had no significant effect on the MIC (ANOVA, F_7, 420_ = 1.977, *p* = 0.06).

## Discussion

This study has identified viable *Candida* spp. on environmental plastic pollution collected from both freshwater and marine areas. The five species detected on plastic pollution (*C. glabrata*, *C. pseudolambica*, *C. sojae*, *C. tropicalis* and *P. kudriavzevii*) were all pathogenic, thermotolerant and resistant to at least one antifungal drug, with wastewater discharge acting as a clear point source for environmental contamination with drug resistant *C. glabrata* and *C. tropicalis*.

Most species of *Candida* were isolated from plastics in more than one of the aquatic environments (i.e., marine, estuarine, and freshwater), suggesting a high level of adaptability to environmental stress. However, the protective environment afforded by the plastisphere can also increase tolerance to environmental stressors and facilitate survival of microorganisms as plastics move through the freshwater-marine continuum. *Candida* are one of the most dominant genera in WWTPs (Assress et al. 2019), and it is here that they can come into contact with plastic pollutants (e.g., wet wipes) before being released into the environment. *C. tropicalis*, *C. glabrata* and *P. kudriavzevii* were all detected directly on plastics placed next to an effluent pipe. *C. tropicalis* isolated from wet wipes at a marine site had higher levels of antifungal resistance compared to isolates from the other plastic types, and hospital effluents may provide an additional risk of releasing drug-resistant species of pathogenic *Candida*, e.g., *C. auris,* into the environment (Mataraci-Kara et al. 2020). In a clinical setting, the potential for biofilm formation by *Candida* on plastic surfaces can be influenced by the polymer type (Estivill et al. 2011); however, in general, the type of plastic that environmental isolates of *Candida* were colonising had little influence on their subsequent virulence, thermotolerance, or drug resistance.

*Candida albicans* may also enter surface water through livestock faeces and subsequent agricultural run-off (Maneenil et al. 2015; Seyedmousavi et al. 2018). The ability of *C. albicans* isolates to bind to plastic in the environment could increase the chances of human contact, potentially representing a novel zoonotic disease transmission pathway. However, although *Candida albicans* was detected on plastics deployed in the river upstream of the effluent pipe, it was only detected once downstream of the pipe and was not detected on any of the plastic pollution collected at sites A–F, which may reflect the poor competitive capability of *C. albicans* in the plastisphere.

Adhesion strategies are widely recognised as major virulence factors in pathogenic *Candida*, with several species possessing GPI-modified cell wall proteins (Sundstrom 2002). The degree of adhesion varies between species, with *C. tropicalis* and *P. kudriavzevii* demonstrating greater adhesion than *C. glabrata*. Binding to environmental plastic surfaces has previously been demonstrated to increase the virulence of fungal pathogens (Gkoutselis et al. 2024), but whether biofilm formation on environmental plastic surfaces increases the expression of adhesion factors of environmental isolates of *Candida* and whether this influences subsequent mechanisms of pathogenicity is unknown.

*C. glabrata* and *C. tropicalis* were the most virulent species isolated from environmental plastic wastes. Invasive candidiasis caused by *C. glabrata* causes 20–50% mortality at 30 days, and *C. tropicalis* is responsible for 7% of candidiasis infections, with mortality as high as 60% in adults (WHO, 2022). Whilst *C. albicans* is a common member of the human microbiota, survival on surfaces outside of the human body is limited compared to other *Candida* species (Wißmann et al. 2021). *C. albicans,* which is responsible for 65% of candidiasis infections, was detected upstream of the effluent discharge pipe, but was not isolated from any of the other plastic wastes collected at Sites A–F. Globally, the proportion of *C. albicans* infections is decreasing in parallel with an increasing incidence of *C. glabrata* and *C. tropicalis* (Berkow and Lockhart 2017), which highlights the importance of quantifying the environmental reservoirs and life-cycle of non-*albicans* species of pathogenic *Candida*.

Human body temperature is a potent nonspecific defence against fungal pathogens, above which opportunistic fungi are usually unable to grow and establish an infection. Several of the environmental isolates of *Candida* in this study were able to grow at 38 °C, indicating that they may be capable of overcoming the mammalian thermal defence barrier. After simulating the transfer of *Candida* from the environment to the human body (i.e., initial incubation at 18 °C and then moved to 38 °C), most environmental isolates of *Candida* showed robust growth. The highest growth rates were demonstrated by *P. kudriavzevii*, indicating that it was capable of adapting quickly to a significant temperature change, e.g., by shifting the metabolic pathways from glycerol to trehalose synthesis (Liu et al. 2005). Similarly, *C. glabrata* can tolerate high temperatures due to changes in the calcineurin pathways (Chen et al. 2012). Importantly, several factors that facilitate thermotolerance (e.g., heat-shock protein 90 and calcineurin) can also enhance antifungal resistance (Berman and Krysan 2020). This is particularly concerning, with rising environmental temperatures as a result of climate change, which could facilitate the development of more resistant and harmful strains of *Candida* (Salazar-Hamm & Torres-Cruz, 2024; Williams et al. 2024).

Less growth was seen when isolates were initially incubated at 38 °C before incubation at 18 °C (representing the movement of *Candida* from the human body to the environment), indicating lower levels of survival in the environment. However, the protective environment provided by the plastisphere may increase survival of fungi at these temperatures (Lacerda et al. 2020). For all environmental isolates, growth at 28 °C was higher, indicating that in warmer locations *Candida* is more likely to survive the transition from the human body to the environment.

*C. sojae* is an emerging pathogen, and despite its physiological similarities to *C. albicans* and *C. tropicalis,* it was only in 2022 that the first human infection was reported, most likely due to being previously misidentified (Chrenkova et al. 2022). However, in this study, *C. sojae* had lower levels of virulence and antifungal resistance compared to the other species, implying that this species presents a lower risk to public health. There are currently no reports of *C. pseudolambica* causing human infection, although the isolates recovered from environmental plastics did have high levels of virulence in the *G. mellonella* model. This species is also closely related to pathogenic *P. kudriavzevii* (Ebadi et al. 2022), indicating that there is the evolutionary potential for this species to emerge as a human pathogen. However, *C. pseudolambica* and *C. sojae* showed less growth at 38 °C, and both species have lower maximum growth temperatures (below 40 °C) compared to other *Candida* species (Ebadi et al. 2022), indicating that temperature may be the main barrier against infection by these two species.

Globally, pathogenic fungi are evolving resistance to all licensed antifungal drugs (Fisher et al. 2022). Azoles, especially fluconazole, are the most widely used antifungals because of their high effectiveness, low toxicity and ability to be taken orally (Partha et al. 2022). However, the environmental isolates screened in this study had high levels of resistance to fluconazole, with several isolates resistant to concentrations of 64 mg/L. Despite *C. albicans* being fairly susceptible to fluconazole, other species of *Candida* such as *C. tropicalis* and *C. glabrata* have relatively high rates of fluconazole resistance. For example, in South Korea fluconazole resistance in *C. glabrata* increased from 0 to 8.3% between 2008 and 2018 (Won et al. 2021), whereas *C. auris* can be highly drug resistant, with 93% of isolates resistant to fluconazole (Lockhart et al 2017). Although yet to be isolated from environmental plastic pollution, *C. auris* has already been identified in WWTP effluents (Barber et al. 2023) and can persist and remain pathogenic in the plastisphere in simulated environments (Akinbobola et al. 2024).

Voriconazole is an alternative azole, used to treat candidiasis and fluconazole resistant strains of *Candida*; however, there is concern that voriconazole resistance can emerge following exposure to fluconazole, particularly in cases involving *C. glabrata* (Pfaller et al. 2011). Polyenes and echinocandins are more recently developed antifungals, with higher treatment success rates than azoles (Tashiro et al. 2020). Despite its toxic potential and common side-effects (e.g., nephrotoxicity), amphotericin B is an effective antifungal treatment against progressive and drug-resistant fungal infections with low resistance rates compared to azoles and other antifungals (Cavassin et al. 2021). However, resistance to all anti-fungal drugs is increasing in *Candida*, with evidence of an increase in multi-drug resistance. There are also eco-evolutionary links between environmental and clinical resistance due to the increasing use of agricultural fungicides (particularly azoles), which have been hypothesised to drive increased resistance of *Candida* to antifungal drugs in clinical settings (Fisher et al. 2022).

## Conclusion

Species of *Candida*, including several high priority species on the WHO ‘Fungal priority list’, were isolated from environmental plastic pollution from marine, estuarine, and freshwater ecosystems. Most of these isolates were virulent, thermotolerant, and drug resistant. This highlights a potential public health risk and the urgent need to improve public awareness, monitoring, and environmental management to prevent human exposure to plastic pollution colonised by *Candida*. Climate change and increases in temperature are likely to accelerate the development of resistance and pathogenicity of *Candida*, further highlighting the need for action*.* Plastics placed downstream of a wastewater effluent pipe were rapidly colonised by several *Candida* species, suggesting a potential point source for human pathogenic *Candida* to be introduced into the environment. With an increase in the prevalence of global infections caused by non-albicans species of *Candida*, it is vital that we increase our focus on these emerging and recently emerged pathogens and continue to improve our understanding of environmental reservoirs and subsequent transfer routes to humans.

## Supplementary Information

Below is the link to the electronic supplementary material. 

ESM1 (PDF 1.86 MB)

## Data Availability

All datasets are available in the Environmental Information Data Centre repository (EIDCHELP-81892), https://catalogue.ceh.ac.uk/documents/4477f77c-c1c9-44e4-baeb-3353954c8355

## References

[CR1] Akinbobola AB, Kean R, Hanifi SMA, Quilliam RS (2023) Environmental reservoirs of the drug-resistant pathogenic yeast *Candida auris*. PLoS Pathog 19(4):e1011268 37053164 10.1371/journal.ppat.1011268 PMC10101498

[CR2] Akinbobola A, Kean R, Quilliam RS (2024) Plastic pollution as a novel reservoir for the environmental survival of the drug-resistant fungal pathogen *Candida auris*. Mar Pollut Bull 198:115841 38061145 10.1016/j.marpolbul.2023.115841

[CR3] Arendrup, M.C., Meletiadis, J., Mouton, J.W., Lagrou, K., Hamal, P., Guinea, J., the Subcommittee on Antifungal Susceptibility Testing (AFST) of the ESCMID European Committee for Antimicrobial Susceptibility Testing (EUCAST) (2020) ‘EUCAST DEFINITIVE DOCUMENT E.DEF 7.3.2 Method for the determination of broth dilution minimum inhibitory concentrations of antifungal agents for yeasts’.

[CR4] Arora P, Singh P, Wang Y, Yadav A, Pawar K, Singh A, Padmavati G, Xu J, Chowdhary A (2021) Environmental isolation of *Candida auris* from the coastal wetlands of Andaman Islands, India. Mbio 12(2):3181 10.1128/mBio.03181-20 PMC8092279 33727354

[CR5] Assress HA, Selvarajan R, Nyoni H, Ntushelo K, Mamba BB, Msagati TA (2019) Diversity, co-occurrence and implications of fungal communities in wastewater treatment plants. Sci Rep 9(1):14056 31575971 10.1038/s41598-019-50624-z PMC6773715

[CR6] Baker T, Bester A, Sebolai O, Albertyn J, Pohl C (2024) Biofilms on urban aquatic plastic pollution as a reservoir for pathogenic yeasts. J Water Health 22(10):1826–1842

[CR7] Barber C, Crank K, Papp K, Innes GK, Schmitz BW, Chavez J, Rossi A, Gerrity D (2023) Community-scale wastewater surveillance of *Candida auris* during an ongoing outbreak in southern Nevada. Environ Sci Technol 57(4):1755–1763 36656763 10.1021/acs.est.2c07763 PMC9893721

[CR8] Berkow, E.L. and Lockhart, S.R., (2017) ‘Fluconazole resistance in *Candida* species: a current perspective.’ *Infection and drug resistance*, pp.237–245. 10.2147/IDR.S118892 PMC5546770 28814889

[CR9] Berman J, Krysan DJ (2020) Drug resistance and tolerance in fungi. Nat Rev Microbiol 18(6):319–331 32047294 10.1038/s41579-019-0322-2 PMC7231573

[CR10] Bongomin F, Gago S, Oladele RO, Denning DW (2017) Global and multi-national prevalence of fungal diseases—estimate precision. Journal of Fungi 3(4):57 29371573 10.3390/jof3040057 PMC5753159

[CR11] Brandao LR, Medeiros AO, Duarte MC, Barbosa AC, Rosa CA (2010) Diversity and antifungal susceptibility of yeasts isolated by multiple-tube fermentation from three freshwater lakes in Brazil. J Water Health 8(2):279–289 20154391 10.2166/wh.2009.170

[CR12] Carvalho A, Costa-De-Oliveira S, Martins ML, Pina-Vaz C, Rodrigues AG, Ludovico P, Rodrigues F (2007) Multiplex PCR identification of eight clinically relevant *Candida* species. Med Mycol 45(7):619–627 17885953 10.1080/13693780701501787

[CR13] Cavassin FB, Baú-Carneiro JL, Vilas-Boas RR, Queiroz-Telles F (2021) Sixty years of amphotericin B: an overview of the main antifungal agent used to treat invasive fungal infections. Infectious Diseases and Therapy 10:115–147 33523419 10.1007/s40121-020-00382-7 PMC7954977

[CR14] Chen YS, Yanagida F, Chen LY (2009) Isolation of marine yeasts from coastal waters of northeastern Taiwan. Aquat Biol 8(1):55–60

[CR15] Chen, Y.L., Konieczka, J.H., Springer, D.J., Bowen, S.E., Zhang, J., Silao, F.G.S., Bungay, A.A.C., Bigol, U.G., Nicolas, M.G., Abraham, S.N. and Thompson, D.A., (2012) ‘Convergent evolution of calcineurin pathway roles in thermotolerance and virulence in *Candida glabrata*.’ G3: Genes| Genomes| Genetics 2(6):pp.675–691. 10.1534/g3.112.002279 PMC3362297 22690377

[CR16] Chrenkova V, Vadkertiova R, Vlachova K, Babjuk M, Lischke R, Bebrova E, Hubacek P (2022) *Candida sojae*: First report of a human infection. Journal De Mycologie Medicale 32(4):101309 35870417 10.1016/j.mycmed.2022.101309

[CR17] Denning DW (2024) Global incidence and mortality of severe fungal disease. Lancet Infect Dis 24(7):e428–e438 38224705 10.1016/S1473-3099(23)00692-8

[CR18] Dire O, Ahmad A, Duze S, Patel M (2023) Survival of *Candida auris* on environmental surface materials and low-level resistance to disinfectant. J Hosp Infect 137:17–23 37116661 10.1016/j.jhin.2023.04.007

[CR19] Ebadi T, Najafpour GD, Younesi H, Mohammadi M (2022) Rapid biodegradation of diazinon using a novel strain of *Candida pseudolambica*. Environ Technol Innov 25:102218

[CR20] Ekdahl LI, Salcedo JA, Dungan MM, Mason DV, Myagmarsuren D, Murphy HA (2023) Selection on plastic adherence leads to hyper-multicellular strains and incidental virulence in the budding yeast. Elife 12:e81056 37916911 10.7554/eLife.81056 PMC10764007

[CR21] Erni-Cassola G, Zadjelovic V, Gibson MI, Christie-Oleza JA (2019) Distribution of plastic polymer types in the marine environment; a meta-analysis. J Hazard Mater 369:691–698 30826562 10.1016/j.jhazmat.2019.02.067

[CR22] Estivill D, Arias A, Torres-Lana A, Carrillo-Muñoz AJ, Arévalo MP (2011) Biofilm formation by five species of *Candida* on three clinical materials. J Microbiol Methods 86(2):238–242 21664387 10.1016/j.mimet.2011.05.019

[CR23] Plastics Europe, (2022) ‘Annual production of plastics worldwide from 1950 to 2021.’ [Accessed: Retrieved October 09, 2023].

[CR24] Fisher MC, Alastruey-Izquierdo A, Berman J, Bicanic T, Bignell EM, Bowyer P, Bromley M, Brüggemann R, Garber G, Cornely OA (2022) Tackling the emerging threat of antifungal resistance to human health. Nat Rev Microbiol 20(9):557–571 35352028 10.1038/s41579-022-00720-1 PMC8962932

[CR25] Fisher, M.C., Burnett, F., Chandler, C., Gow, N.A., Gurr, S., Hart, A., Holmes, A., May, R.C., Quinn, J., Soliman, T. and Talbot, N.J., (2024). A one health roadmap towards understanding and mitigating emerging Fungal Antimicrobial Resistance: fAMR. npj Antimicrob Resist 2(1):p.36. 10.1038/s44259-024-00055-2 PMC11543597 39524479

[CR26] Fotedar R, Chatting M, Kolecka A, Zeyara A, Al Malki A, Kaul R, Bukhari SJ, Moaiti MA, Febbo EJ, Boekhout T (2022) Communities of culturable yeasts and yeast-like fungi in oligotrophic hypersaline coastal waters of the Arabian Gulf surrounding Qatar. Antonie Van Leeuwenhoek 115(5):609–633 35322327 10.1007/s10482-022-01722-y

[CR27] Geyer R, Jambeck JR, Law KL (2017) Production, use, and fate of all plastics ever made. Sci Adv 3(7):e1700782 28776036 10.1126/sciadv.1700782 PMC5517107

[CR28] Gkoutselis G, Rohrbach S, Harjes J, Obst M, Brachmann A, Horn MA, Rambold G (2021) Microplastics accumulate fungal pathogens in terrestrial ecosystems. Sci Rep 11(1):13214 34267241 10.1038/s41598-021-92405-7 PMC8282651

[CR29] Gkoutselis G, Rohrbach S, Harjes J, Brachmann A, Horn MA, Rambold G (2024) Plastiphily is linked to generic virulence traits of important human pathogenic fungi. Communications Earth & Environment 5(1):51

[CR30] Gómez-Molero E, De-la-Pinta I, Fernández-Pereira J, Groß U, Weig M, Quindós G, De Groot PW, Bader O (2021) Candida parapsilosis colony morphotype forecasts biofilm formation of clinical isolates. Journal of Fungi 7(1):33 33430377 10.3390/jof7010033 PMC7827155

[CR31] Jiang P, Zhao S, Zhu L, Li D (2018) Microplastic-associated bacterial assemblages in the intertidal zone of the Yangtze Estuary. Sci Total Environ 624:48–54 29247904 10.1016/j.scitotenv.2017.12.105

[CR32] Junaid M, Siddiqui JA, Sadaf M, Liu S, Wang J (2022) Enrichment and dissemination of bacterial pathogens by microplastics in the aquatic environment. Sci Total Environ 830:154720 35337880 10.1016/j.scitotenv.2022.154720

[CR33] Kainz K, Bauer MA, Madeo F, Carmona-Gutierrez D (2020) Fungal infections in humans: the silent crisis. Microbial Cell 7(6):143 32548176 10.15698/mic2020.06.718 PMC7278517

[CR34] Kibria MG, Masuk NI, Safayet R, Nguyen HQ, Mourshed M (2023) Plastic waste: challenges and opportunities to mitigate pollution and effective management. International Journal of Environmental Research 17(1):20 36711426 10.1007/s41742-023-00507-z PMC9857911

[CR35] Lacerda ALDF, Proietti MC, Secchi ER, Taylor JD (2020) Diverse groups of fungi are associated with plastics in the surface waters of the Western South Atlantic and the Antarctic Peninsula. Mol Ecol 29(10):1903–1918 32270556 10.1111/mec.15444

[CR36] Lachance, M.A., Boekhout, T., Scorzetti, G., Fell, J.W. and Kurtzman, C.P., (2011). Candida berkhout (1923). In The Yeasts (pp. 987–1278). Elsevier.

[CR37] Li WC, Tse HF, Fok L (2016) Plastic waste in the marine environment: a review of sources, occurrence and effects. Sci Total Environ 566:333–349 27232963 10.1016/j.scitotenv.2016.05.084

[CR38] Liu HJ, Liu DH, Zhong JJ (2005) Interesting physiological response of the osmophilic yeast *Candida krusei* to heat shock. Enzyme Microb Technol 36(4):409–416

[CR40] Lockhart SR, Etienne KA, Vallabhaneni S, Farooqi J, Chowdhary A, Govender NP, Colombo AL, Calvo B, Cuomo CA, Desjardins CA, Berkow EL (2017) Simultaneous emergence of multidrug-resistant Candida auris on 3 continents confirmed by whole-genome sequencing and epidemiological analyses. Clin Infect Dis 64(2):134–140 27988485 10.1093/cid/ciw691 PMC5215215

[CR41] Maneenil G, Payne MS, Senthamarai Kannan P, Kallapur SG, Kramer BW, Newnham JP, Miura Y, Jobe AH, Kemp MW (2015) Fluconazole treatment of intrauterine *Candida albicans* infection in foetal sheep. Pediatr Res 77(6):740–748 25760552 10.1038/pr.2015.48

[CR42] Mataraci-Kara E, Ataman M, Yilmaz G, Ozbek-Celik B (2020) Evaluation of antifungal and disinfectant-resistant *Candida* species isolated from hospital wastewater. Arch Microbiol 202:2543–2550 32656678 10.1007/s00203-020-01975-z

[CR43] Medeiros AO, Kohler LM, Hamdan JS, Missagia BS, Barbosa FA, Rosa CA (2008) Diversity and antifungal susceptibility of yeasts from tropical freshwater environments in Southeastern Brazil. Water Res 42(14):3921–3929 18678387 10.1016/j.watres.2008.05.026

[CR46] Oliveira MM, Proenca AM, Moreira-Silva E, Dos Santos FM, Marconatto L, de Castro AM, Medina-Silva R (2022) Biochemical features and early adhesion of marine *Candida parapsilosis* strains on high-density polyethylene. J Appl Microbiol 132(3):1954–1966 34787949 10.1111/jam.15369

[CR47] Parslow BY, Thornton CR (2022) Continuing shifts in epidemiology and antifungal susceptibility highlight the need for improved disease management of invasive candidiasis. Microorganisms 10(6):1208 35744725 10.3390/microorganisms10061208 PMC9228503

[CR48] Partha ADSL, Widodo ADW, Endraswari PD (2022) Evaluation of fluconazole, itraconazole, and voriconazole activity on *Candida albicans*: a case control study. Annals of Medicine and Surgery 84:104882 36536737 10.1016/j.amsu.2022.104882 PMC9758354

[CR49] Pfaller MA, Andes D, Arendrup MC, Diekema DJ, Espinel-Ingroff A, Alexander BD, Brown SD, Chaturvedi V, Fowler CL, Ghannoum MA, Johnson EM (2011) Clinical breakpoints for voriconazole and *Candida* spp. revisited: review of microbiologic, molecular, pharmacodynamic, and clinical data as they pertain to the development of species-specific interpretive criteria. Diagn Microbiol Infect Dis 70(3):330–343 21546199 10.1016/j.diagmicrobio.2011.03.002

[CR50] R Core Team (2021). R: a language and environment for Statistical Computing R Foundation for Statistical Computing, Vienna, Austria URL https://www.R-Project.org

[CR51] Romera, D., Aguilera-Correa, J.J., García-Coca, M., Mahillo-Fernandez, I., Viñuela-Sandoval, L., García-Rodríguez, J., Esteban, J., (2020) ‘The *Galleria mellonella* infection model as a system to investigate the virulence of *Candida auris* strains.’ Pathog dis 78(9): p.ftaa067. 10.1093/femspd/ftaa067 33098293

[CR52] Salazar-Hamm P, Torres-Cruz TJ (2024) The impact of climate change on human fungal pathogen distribution and disease incidence. Current Clinical Microbiology Reports 11(3):140–152

[CR53] Sautour M, Lemaître J, Ranjard L, Truntzer C, Basmaciyan L, Depret G, Hartmann A, Dalle F (2021) Detection and survival of *Candida albicans* in soils. Environmental DNA 3(6):1093–1101

[CR54] Seyedmousavi, S., Bosco, S.D.M., De Hoog, S., Ebel, F., Elad, D., Gomes, R.R., Jacobsen, I.D., Jensen, H.E., Martel, A., Mignon, B. and Pasmans, F., (2018). Fungal infections in animals: a patchwork of different situations. Med mycol 56(suppl_1):pp.S165-S187. 10.1093/mmy/myx104 PMC6251577 29538732

[CR55] Silva S, Negri M, Henriques M, Oliveira R, Williams DW, Azeredo J (2011) Adherence and biofilm formation of non-Candida albicans Candida species. Trends Microbiol 19(5):241–247 21411325 10.1016/j.tim.2011.02.003

[CR56] Siscar-Lewin S, Hube B, Brunke S (2022) Emergence and evolution of virulence in human pathogenic fungi. Trends Microbiol 30(7):693–704 35058122 10.1016/j.tim.2021.12.013

[CR57] Sundstrom P (2002) Adhesion in *Candida* spp. Cell Microbiol 4(8):461–469 12174081 10.1046/j.1462-5822.2002.00206.x

[CR58] Tashiro S, Osa S, Igarashi Y, Watabe Y, Liu X, Enoki Y, Taguchi K, Mayumi T, Miyazaki Y, Takesue Y, Matsumoto K (2020) Echinocandins versus non-echinocandins for the treatment of invasive candidiasis: a meta-analysis of randomized controlled trials. J Infect Chemother 26(11):1164–1176 32620421 10.1016/j.jiac.2020.06.008

[CR59] Trost A, Graf B, Eucker J, Sezer O, Possinger K, Göbel UB, Adam T (2004) Identification of clinically relevant yeasts by PCR/RFLP. J Microbiol Methods 56(2):201–211 14744449 10.1016/j.mimet.2003.10.007

[CR60] Wallbank JA, Lear G, Kingsbury JM, Weaver L, Doake F, Smith DA, Audrézet F, Maday SD, Gambarini V, Donaldson L, Theobald B (2022) Into the Plastisphere, where only the generalists thrive: early insights in plastisphere microbial community succession. Front Mar Sci 9:841142

[CR61] WHO (2022) ‘WHO fungal priority pathogens list to guide research, development and public health action’. (World Health Organization) https://www.who.int/publications/i/item/9789240060241

[CR62] Williams SL, Toda M, Chiller T, Brunkard JM, Litvintseva AP (2024) Effects of climate change on fungal infections. PLoS Pathog 20(5):e1012219 38814855 10.1371/journal.ppat.1012219 PMC11139277

[CR63] Wißmann JE, Kirchhoff L, Brüggemann Y, Todt D, Steinmann J, Steinmann E (2021) Persistence of pathogens on inanimate surfaces: a narrative review. Microorganisms 9(2):343 33572303 10.3390/microorganisms9020343 PMC7916105

[CR64] Wójcik A, Kurnatowski P, Błaszkowska J (2013) Potentially pathogenic yeasts from soil of children’s recreational areas in the city of Łódź (Poland). Int J Occup Med Environ Health 26:477–487 24018998 10.2478/s13382-013-0118-y

[CR65] Won EJ, Choi MJ, Kim MN, Yong D, Lee WG, Uh Y, Kim TS, Byeon SA, Lee SY, Kim SH, Shin JH (2021) Fluconazole-resistant *Candida glabrata* bloodstream isolates, South Korea, 2008–2018. Emerg Infect Dis 27(3):779 33624581 10.3201/eid2703.203482 PMC7920659

[CR66] Zettler ER, Mincer TJ, Amaral-Zettler LA (2013) Life in the “plastisphere”: Microbial communities on plastic marine debris. Environ Sci Technol 47:7137–7146 23745679 10.1021/es401288x

