# Supplementary material for: Thermotolerance, virulence, and drug resistance of human pathogenic Candida species colonising plastic pollution in aquatic ecosystems
Source: Environ Sci Pollut Res Int. 2025 Jun 4;32(24):14993–5005. doi: 10.1007/s11356-025-36558-2 (PMC12202632; doi:10.1007/s11356-025-36558-2)
Supplement: Supplementary file 1 — (PDF 1.86 MB) [file 11356_2025_36558_MOESM1_ESM.pdf]

Thermotolerance, virulence, and drug resistance of human pathogenic *Candida* species colonising plastic pollution in aquatic ecosystems

Rebecca Metcalf[1], Ayorinde Akinbobola[1], Luke Woodford[1], Richard S. Quilliam[1]*

[1]Biological and Environmental Sciences, Faculty of Natural Sciences, University of Stirling, Stirling, FK9 4LA, UK

***Corresponding author email**: Richard Quilliam, richard.quilliam@stir.ac.uk

Abstract

Recently, five species of the pathogenic yeast *Candida* have been classified as priority fungal pathogens by the World Health Organisation (WHO), yet the persistence and niches of pathogenic species of *Candida* in the environment remains unclear. However, despite the ability of *Candida* to form strong biofilms on plastics in clinical settings, the potential for plastics in the environment to facilitate survival and dissemination of *Candida* as has not been determined. Here, we have collected plastic pollutants from freshwater, estuarine, and marine environments and successfully isolated five pathogenic species of *Candida*, including three that are on the WHO Fungal Priority Pathogens list (*C.*

glabrata, C. tropicalis, Pichia kudriavzevii (formerly known as *Candida krusei*). All environmental isolates were resistant to at least one antifungal drug, thermotolerant to human body temperature, and showed pathogenicity in a *Galleria mellonella* model of infection. In addition, polyethylene (PE) plastic pellets were deployed upstream and downstream of a wastewater discharge pipe in a small freshwater stream. Although all pellets became colonised by pathogenic species of *Candida*, there were clear differences in species prevalence upstream and downstream of the effluent, indicating a clear point source for *Candida* discharge into surface waters. The global incidence of candidiasis, particularly by drug resistant strains, is increasing, and it is therefore critical that we increase our focus on the environmental reservoirs of these pathogens and continue to improve our understanding of their environmental persistence and subsequent transfer routes to humans.

Keywords: Antifungal resistance; Environmental pollution; Fungal pathogens; Plastisphere; Pathogenic yeast

~~1.~~ Introduction

Plastic pollution in the environment is of major global concern, with an estimated 390 million metric tonnes of plastic being produced annually, and production predicted to double in the next 20 years (Plastics Europe 2022). Only a small proportion of the total plastic produced globally is recycled, which results in large quantities of plastic waste ending up in the environment (Geyer et al. 2017; Kibria et al. 2023). Plastics are inherently recalcitrant to degradation and, as such, plastic pollutants can negatively impact both terrestrial and aquatic ecosystems (Li et al. 2016; Kibria et al. 2023). Importantly, once in the environment, plastic surfaces become rapidly colonised by microbial biofilm that comprised of complex

microbial communities (Zettler et al., 2013). Such ‘plastisphere’ communities can contain (or even enrich) human pathogens, which can then be disseminated within different environmental matrices (Junaid et al., 2022). The majority of plastisphere research has focussed on prokaryotic communities, including human bacterial pathogens (Jiang et al., 2018); however, eukaryotic microorganisms, including human fungal pathogens can also associate with the plastisphere (Gkoutselis et al., 2021).

Millions of severe infections and deaths are caused annually by fungal pathogens (Denning, 2024; Kainz et al., 2020). One of the most important fungal infections is invasive candidiasis, caused by several species of the yeast *Candida*, with an estimated 700,000 cases annually (Bongomin et al., 2017). Candidiasis also includes less severe cutaneous and mucosal infections, such as thrush. Globally, infections due to potentially pathogenic *Candida* species are increasing, due in part to the emergence of more virulent strains of *Candida* (Siscar-Lewin *et al.,* 2022), which is further compounded by the simultaneous emergence of pathogenic strains of drug-resistant *Candida* (Fisher et al., 2022; Parslow and Thornton, 2022). This has led the WHO to add six species of *Candida* to the Fungal Pathogen Priority list (WHO, 2023), i.e., *Candida* (*Candidozyma*) *auris* and *C. albicans* both classed as Critical priority; *C. glabrata* (*Nakaseomyces glabrata*), *C. parapsilosis,* and *C. tropicalis,* all classed as High priority; and *C. krusei* (*Pichia kudriavzevii*) classed as Medium priority.

Understanding the source of infection and interaction of fungal pathogens within the environment is important for the effective management of fungal infections (Fisher et al., 2024), particularly as *Candida* is notorious for forming strong biofilms on plastic surfaces in healthcare settings (Estivill et al., 2011). Most of the approximately 200 species of *Candida* are non-pathogenic and persist in the environment, e.g., in water and soil, or associated with food, plants or insects

(Lachance et al. 2011). However, several species are opportunistically pathogenic, taking advantage of the opportunity to colonise humans and cause disease (Ekdahl et al., 2023). *Candida* can survive the transition through wastewater treatment plants (WWTPs) (Assress et al., 2019) and persist in various environmental matrices (e.g., coastal wetlands (*C. auris*), freshwater (*C. glabrata, P. kudriavzevii, C. tropicalis*), seawater (*C. albicans, C. tropicalis*), and soil (*C. albicans, C. glabrata*; *C. tropicalis*) (Akinbobola et al., 2023; Arora *et al.,* 2021; Brandao *et al.,* 2010; Chen *et al.,* 2009; Medeiros *et al.,* 2008; Fotedar *et al.,* 2022; Wójcik et al. 2013, Sautour *et al.,* 2021). Therefore, during their transition through WWTPs and the environment, pathogenic species of *Candida* could encounter and colonise environmental plastic pollution.

Like other yeast species, *Candida* spp. are known to possess several features and mechanisms (e.g., epithelial adhesin expression, hydrolytic enzyme secretion) which enable them to successfully adhere and form biofilms (Silva et al., 2011). *C. parapsilosis* readily forms biofilm on plastics in clinical settings (Gomez-Molero et al., 2021), and previous studies with *C. auris* and *C. parapsilosis* have demonstrated that these species can also adhere to plastic surfaces under environmental mesocosm conditions (Oliveira et al., 2022; Dire et al., 2023; Akinbobola et al., 2024); whilst ITS sequencing has shown that *Candida* spp. are present in the plastisphere (Baker et al., 2024; Wallbank et al., 2022). Therefore, plastic pollutants could constitute an important vehicle for the dissemination of human pathogenic *Candida* in the environment with significant implications for the epidemiology and environmental management of *Candida* infections. In order to determine the co-pollutant risk of plastic pollutants to human health, we have collected and screened different types of plastic pollution in marine, estuarine, and freshwater environments for pathogenic species of *Candida*, and assessed these isolates for their thermotolerance, anti-fungal drug resistance, and their

pathogenicity in a *Galleria* model of infection. Additionally, we have investigated the potential for wastewater discharge to act as a point source for the introduction of pathogenic *Candida* into surface waters by deploying cages containing polyethylene (PE) plastic pellets upstream and downstream of an effluent pipe discharging from a WWTP, and screening for subsequent colonisation by pathogenic species of *Candida*.

~~2.~~ Materials and methods

~~2.1.1~~ *Sample collection and processing*

Plastic debris was collected from six sites (A–F) on three separate days (March–May 2023; Fig. 1; Table S1). The sites included marine, estuarine, and freshwater beaches on the east (Firth) and west (Clyde) catchments in central Scotland, UK. Two of the beaches sampled were designated bathing water beaches, regulated under the EU Bathing Water Directive (BWD) 2006/7/EC. Central Scotland contains several large urban centres (e.g., Glasgow, Edinburgh) and has the highest population density in Scotland (population 4.2 million). At each site, items of plastic pollution were collected using sterile forceps and placed into sterile Ziplock bags. All samples were stored at 4°C and processed within 24 h. Plastic samples were sorted into composite samples of either ‘hard plastics’ (High density polyethylene [HDPE] and Polyethylene terephthalate [PET]) such as cotton bud sticks, tampon applicators, and plastic fragments; ‘soft plastics’ (Low-density polyethylene [LDPE], Polypropylene [PP] and Polyethylene (PE) such as plastic bags, food wrappers, wet wipes); or ‘polystyrene’ (PS).

~~2.1.2~~ *Point source discharge of Candida in receiving waters~~.~~*

To determine the role of wastewater discharge for introducing pathogenic *Candida* species into the environment, 4-–mm PE beads (Goodfellow, UK) were placed in sterile stainless-steel cages in large metal baskets upstream (site G) and downstream (site H) of a waste-water treatment plant (WWTP) effluent pipe (Table S1) in May 2024. PE was chosen for this experiment as it is the most commonly produced plastic polymer globally (Erni-Cassola et al., 2019). The metal baskets were suspended in the river using fishing line approximately 15 metres upstream of, and directly next to the WWTP effluent pipe. Plastic pellets were recovered from the baskets at days 1, 4, and 8, placed in sterile Ziplock bags, and processed on the same day as collection.

2.2 *Recovery of Candida spp. using selective media*

Different quantities of plastic types were collected from sites A-–F. Replicate composite samples of each plastic type (13 hard plastics (e.g., PET); 13 soft plastics (e.g., PE); 1 polystyrene; 1 wet wipe; 1 cotton bud stick) from each site were pre-enriched in 100-—ml yeast extract peptone dextrose (YPD) broth supplemented with antibiotics (gentamicin 50 mg/L, chloramphenicol 50 mg/L; Sigma-Aldrich, USA) and incubated at 30°C for 48 h in a shaking incubator (120 rpm; Incu-Shake MIDI Benchtop Shaking Incubator, SciQuip, UK). After incubation, 100 µl of the overnight culture and a 1:1000 diluent of the same culture was separately spread onto Sabouraud glucose agar with 50 mg/L chloramphenicol (SGA; Sigma-Aldrich, USA) supplemented with gentamicin 50 mg/L, and fluconazole at three different concentrations (0, 16, 64 mg/L) to select for fluconazole sensitive, moderately resistant and resistant strains. Plates were inverted and incubated for 48 h (30°C). Colonies were then selected based on different morphologies on Sabouraud glucose agar and streaked onto

CHROMagar™ *Candida* Plus agar (CHROMagar™, France) and incubated (30°C, 48 h). *Candida* species were presumptively identified based on colony colour and morphology on CHROMagar™ *Candida* Plus agar. Twenty-seven colonies were isolated, and glycerol stocks (final concentration 40% glycerol) prepared and frozen at −20°C.

Cages collected from sites G and H were washed in 100 ml MilliQ water (Millipore) by rinsing three times. Fifty pieces of plastic (four independent replicates) were removed from each cage and placed in a sterile 30 ml glass universal with 5 ml of pre-enrichment broth of YPD, supplemented with 50 mg/L of chloramphenicol and gentamycin (Sigma-Aldrich, USA), and incubated on a shaker at 37°C for 48 h. Following incubation, serial dilutions of each broth were performed using sterile PBS, then 100 µl of 10^{-3} and 10^{-5} dilutions were spread on CHROMagar *Candida* Plus agar (CHROMagar™, France). Plates were incubated (37°C for 48 h) and single colonies showing morphological characteristics of *Candida* species were isolated for PCR screening, sanger sequencing and minimum inhibitory concentration testing. The single colonies were picked and mixed in 1:1 YPD and 80% glycerol and stored at −80°C.

2.2 *Characterisation of Candida isolates from environmental plastic pollution*

2.2.1 ***PCR and sequencing for Candida identification***

Glycerol stocks of 27 selected isolates were grown overnight in 5 ml YPD (120 rpm, 37°C, 24 h). Colony PCR was carried out with primers from Carvalho et al. (2007) targeting the ITS region to identify the five common pathogenic species of *Candida*, i.e., *C. albicans*, *C. glabrata*, *P. kudriavzevii*, *C. parapsilosis*, and *C. tropicalis*. Amplification reactions consisted of 12.5 µL multiplex master mix (New England Biolabs, UK), 1 µL each of the forward and reverse primers (10 µmol/L), 2

µL of each DNA sample and 8.5 µL of sterile water in a final reaction volume of 25 µL. PCR amplification was carried out in a thermal cycler (Veriti 96-well thermal cycler, Applied Biosystems, USA) using the following cycle: 10 min initial denaturation at 94°C, followed by 40 cycles of 94°C for 15 s, 55°C for 30 s and 65°C for 45 s, with a final extension of 65°C for 10 min. All PCR products were run down a 2% agarose gel using GelRed® staining (Biotium, USA) and visualised under UV. Different amplicon sizes were used to differentiate between distinct *Candida* species: *C. albicans* (446 bp), *C. glabrata* (839 bp), *P. kudriavzevii* (169 bp), *C. parapsilosis* (370 bp) and *C. tropicalis* (507 bp).

To further confirm the identity of each isolate, the ITS1 region was amplified and sequenced. Additionally, a selection of suspected *Candida* colonies from Sites G and H were selected for screening. Firstly, DNA was extracted using DNeasy Tissue kit (Qiagen, Germany) and eluted in 200 µL Buffer AE, and primers from Trost et al. (2004) used to amplify the ITS1 region (Forward primer: 5′-GTCAAACTTGGTCATTTA-3′; Reverse primer: 5′-TTCTTTTCCTCCGCTTATTG-3′). Amplification reactions consisted of 2x *taq* PCR master mix (Qiagen, Germany), 0.4 µM primer and 5 µL of each DNA sample in a final reaction volume of 25 µL. PCR amplification comprised an initial denaturation step at 94°C for 3 min followed by 34 cycles of amplification (94°C for 30 s, 50°C for 30 s and 72°C for 1 min) followed by a final extension at 72°C for 10 min. PCR products were purified using a QIAquick PCR Purification kit (Qiagen, Germany), and eluted in 50 µL of elution buffer. All purified PCR products underwent Sanger sequencing using Applied Biosystems 3730 DNA analysers (DNA Sequencing and Services, Dundee, UK). Species ID was confirmed using NCBI’s Basic Local Alignment Search Tool (BLAST; NCBI, USA).

2.2.2 *Pathogenicity*

To determine virulence, each isolate was introduced into a *Galleria mellonella* model of infection (Romera et al., 2020). Healthy larvae of *G. mellonella* (Greater wax moth; Livefood, UK) between 2.0 ~~to~~ and 2.5 cm in length were selected, kept in darkness at 15°C and used within one week of purchase. Glycerol stocks of each *Candida* spp. isolate were cultured on SGA agar (37°C, 24 h), and distinct colonies selected and grown in 5-ml YPD (37°C, 24 h, 120 rpm). To ensure that cells were in their exponential growth phase when injected into *G. mellonella*, 1-ml overnight cultures were added to 5-ml YPD and grown to an OD_{570} of 0.7. Cells were then centrifuged (4000 rpm, 4 min) before being washed and resuspended in PBS. Replicate groups of 10 larvae were injected with 10 µL of *Candida* cells (10^5 CFU/larvae) into the hemocoel via the last right pro-limb using a sterile 100-µL Hamilton syringe (Bonaduz, Switzerland) with a 0.6 × ~~x~~ 30 mm needle. All experiments were conducted in biological triplicate. Needles were flushed with ethanol followed by PBS to sterilise them between samples. An inoculation of 10-µL PBS was used as a control to account for mortality caused by physical injury or infection by contamination. Following injection, larvae were incubated at 37°C, and survival evaluated every 24 h for a total of 120 h. Larvae were considered dead when they did not respond to a touch stimulus.

2.2.3 *Thermotolerance*

To determine the thermotolerance profile, each isolate underwent an initial incubation at 18°C (simulating an environmental temperature) or 38°C (simulating an extreme of human body temperature) for 24 h, before being transferred to one of three different temperatures (18, 28 or 38°C) where their growth was measured. Colonies were selected from SGA plates and grown overnight in 5-ml YPD (37°C,

24 h, 120 rpm), and cells centrifuged (4000 rpm, 8 min), washed and resuspended in phosphate buffered saline (PBS). Cell concentrations were adjusted by dilution in sterile distilled water to give a final concentration of approximately 10^5 CFU/ml (PBS serial dilutions were plated on SGA agar and incubated for retrospective enumeration). Cells (20 ml) of each isolate were added to 180 ml YPD broth in 96-well plates (*n* = 3), and plates incubated at either 18, 28 or 38°C for 24 h. I-Button temperature logger chips (iButtonLink, WI, 176 USA) were placed into each incubator to monitor the temperature throughout incubation. Absorbance at 570 nm was measured before and after incubation in a spectrophotometer (Infinite M200 plate reader; Tecan, Switzerland) to determine growth of the isolates.

~~2.2.4~~ ***Antifungal drug resistance***

Each *Candida* isolate, including those isolated from site G and H, was subjected to minimum inhibitory concentration (MIC) analysis to determine antifungal resistance following the European Committee on Antimicrobial susceptibility testing (EUCAST) antifungal MIC method for yeasts (Arendrup et al., 2020). Resistance to four antifungals at ten concentrations was examined: amphotericin B (0.008–4 mg/L), caspofungin (0.008–4 mg/L), fluconazole (0.125–64 mg/L), voriconazole (0.008–4 mg/L) (Thermoscientific, USA). Briefly, 96-well plates were filled with 100 µL double strength RPMI 1640 (2% glucose, Sigma-Aldrich, USA) containing the different concentrations of antifungal drugs. Distinct colonies were selected from SGA plates and grown overnight in 5 ml YPD (37°C, 24 h, 120 rpm). Cells were recovered by centrifugation (4000 rpm, 8 min), washed and resuspended in PBS, and the concentration adjusted to approximately 10^5 cells/ml. Adjusted cultures (100 µL per well) of each isolate were added to 96-well plates and incubated without agitation at 35°C for 24 h. Control wells contained sterile

drug-free medium, with 100 µL of the same adjusted cultures. Absorbance at 530 nm was measured in a spectrophotometer (Infinite M200 plate reader; Tecan, Switzerland) to determine growth of the isolates. The MIC of amphotericin B was determined as the lowest concentration giving rise to an inhibition of ≥90% of growth compared to the drug-free control. The MIC of caspofungin, fluconazole and voriconazole was determined as the lowest concentration giving inhibition of ≥50% growth compared to the drug-free control.

~~2.3~~ *Statistical analysis*

Statistical analysis was conducted in R Studio version 3.3.2 (R Core Team, 2016). Student's *t*-tests and analysis of variance (ANOVA) were used to test for differences in pathogenicity, thermotolerance, and antifungal resistance. All data were tested for distribution and homogeneity of variance (Shapiro-Wilk and Levene's) before parametric tests were used. Where assumptions were not met, data were either log transformed, or non-parametric Mann-Whitney *U* or Kruskal-Wallis tests used. Tukey's and Fisher's LSD post-hoc tests were used to compare groups. Data is reported as mean ± SE, and *P* values ≤0.05 are considered significant.

~~3.~~ Results

~~3.1~~ *Recovery of Candida from environmental plastic pollution*

Five species of *Candida* (*C. glabrata*, *C. pseudolambica*, *C. sojae*, *C. tropicalis* and *P. kudriavzevii*) were isolated from the surfaces of plastic pollutants at sites A-F (Table 1), with three of these species (*C. glabrata*, *C. tropicalis* and *P. kudriavzevii*) listed on the WHO 'Fungal Priority Pathogens' list (WHO, 2022). Twenty isolates of *C. glabrata* were isolated from seven different sites, which

included all three environments, i.e., marine, estuarine, and freshwater. In contrast, most (7/11) *C. tropicalis* isolates were detected on plastics at one of the marine sites. *P. kudriavzevii* was only detected on plastic in freshwater and estuarine sites in the Clyde catchment; both these isolates showed the most similarity to the same accession number (OW988737) despite being isolated from two geographically different sites. *Candida* species were also isolated from the plastic particles deployed at site G, upstream of the effluent pipe (*C. albicans* and *C. glabrata*) and site H, downstream of the effluent pipe, (*C. albicans*, *C. glabrata*, *C. tropicalis* and *P. kudriavzevii*).

~~3.2~~ *Pathogenicity*

All isolates were pathogenic in the *Galleria* larvae infection model, although this varied between the different isolates (Fig. 2). At 120 h after pathogen challenge, the percentage survival of *Galleria* larvae was significantly lower for all isolates compared to the PBS control (Fig. 2; Mann-Whitney *U*, W = 64, $p < 0.01$). The site where *Candida* had been isolated from had no significant influence on the percentage survival of challenged larvae at 120 h (ANOVA, $F_{5, 75} = 0.562$, $p = 0.729$); whilst species of *Candida* did have an effect on percentage survival at 120 h (ANOVA, $F_{4, 76} = 3.223$, $p < 0.05$), although the only significant difference was between *C. sojae* and *C. tropicalis* (Fisher's LSD; difference in means = 42.4%).

~~3.3~~ *Thermotolerance*

Growth of environmental isolates of *Candida* was~~ere~~ significantly affected by temperature (ANOVA, $F_{2, 177} = 516.9$, $p < 0.001$), with growth at 28 and 38°C significantly higher than at 18°C (Fig. 3). The isolates with the lowest levels of growth at 18°C had initially been incubated at 38°C (Fig. 3D), whereas previous

acclimatisation at 18°C produced higher rates of growth (Fig. 3A). In general, there was a significant effect on the rate of growth between *Candida* species (ANOVA, $F_{5, 174} = 2.401$, $p < 0.05$); however, the only significant difference in growth was between *C. glabrata* and *C. pseudolambica* (Fisher's LSD; difference in means = 0.48). Two isolates (e.g., 17, *C. pseudolambica*; and 8, *C. sojae*) had low levels of growth at 38°C (regardless of the pre-incubation temperature), suggesting that these species have a lower optimal growth temperature than the other *Candida* species, which are known to be opportunistic human pathogens. In contrast, growth of *P. kudriavzevii* (i.e., isolates 7 and 16) at 38°C following an initial incubation at 18°C had the highest levels of growth, suggesting that these environmental isolates could successfully acclimatise to the temperature of the human body. The nature of the sites where *Candida* species were isolated from had no significant influence on their growth profile or thermotolerance (ANOVA, $F_{6, 173} = 0.109$, $p = 0.995$).

~~3.4~~ *Antifungal resistance*

All forty *Candida* isolates showed resistance to at least one of the four antifungals tested (amphotericin B, caspofungin, fluconazole, voriconazole), with a single isolate of *C. glabrata* from site C having high levels of resistance to all four antifungals (Fig. 4). However, isolates of *C. tropicalis* expressed a range of resistance profiles to fluconazole, i.e., three of the eleven isolates were resistant to concentrations of 64 mg/L, whilst the other eight isolates were only resistant to concentrations between 0.8 and 8 mg/L (Fig 4C). A similar pattern was observed for voriconazole, where ten of the eleven *C. tropicalis* isolates were highly susceptible (Fig 4D). Two isolates of *C. tropicalis* (site E, samples 21 and 22) which showed high levels of resistance to fluconazole were both isolated from wet wipes, whilst the other strains of *C. tropicalis* from sites E and F were

isolated from hard plastic (Table 1). The *C. albicans* isolates from site G (samples 28-30) showed high susceptibility to fluconazole and voriconazole and typically had greater drug susceptibility than the other *Candida* strains isolated from the effluent pipe at site H. Species identity had an influence on the MIC (ANOVA, $F_{5,422} = 2.882$, $p < 0.05$);, however, the only significant difference was between *C. glabrata* and *C. albicans* ($p = 0.03$). Overall, the location of site the *Candida* had been isolated from had no significant effect on the MIC (ANOVA, $F_{7, 420} = 1.977$, $p = 0.06$).

~~4.~~ Discussion

This study has identified viable *Candida* spp. strains on environmental plastic pollution collected from both freshwater and marine areas. The five species detected on plastic pollution (*C. glabrata*, *C. pseudolambica*, *C. sojae*, *C. tropicalis* and *P. kudriavzevii*) were all pathogenic, thermotolerant and resistant to at least one antifungal drug, with wastewater discharge acting as a clear point source for environmental contamination with drug resistant *C. glabrata* and *C. tropicalis*.

Most species of *Candida* were isolated from plastics in more than one of the aquatic environments (i.e., marine, estuarine, and freshwater), suggesting a high level of adaptability to environmental stress. However, the protective environment afforded by the plastisphere can also increase tolerance to environmental stressors and facilitate survival of microorganisms as plastics move through the freshwater-marine continuum. *Candida* are one of the most dominant genera in WWTPs (Assress et al., 2019), and it is here that they can come into contact with plastic pollutants (e.g., wet wipes) before being released into the environment. *C. tropicalis*, *C. glabrata* and *P. kudriavzevii* were all

detected directly on plastics placed next to an effluent pipe. *C. tropicalis* isolated from wet wipes at a marine site had higher levels of antifungal resistance compared to isolates from the other plastic types, and hospital effluents may provide an additional risk of releasing drug-resistant species of pathogenic *Candida*, e.g., *C. auris,* into the environment (Mataraci-Kara et al., 2020). In a clinical setting, the potential for biofilm formation by *Candida* on plastic surfaces can be influenced by the polymer type (Estivill et al., 2011); however, in general, the type of plastic that environmental isolates of *Candida* were colonising had little influence on their subsequent virulence, thermotolerance, or drug resistance of the *Candida* isolates.

Candida albicans may also enter surface water through livestock faeces and subsequent agricultural run-off (Maneenil et al., 2015; Seyedmousavi et al., 2018). The ability of *C. albicans* isolates to bind to plastic in the environment could increase the chances of human contact, potentially representing a novel zoonotic disease transmission pathway. However, although *Candida albicans* was detected on plastics deployed in the river upstream of the effluent pipe, it was only detected once downstream of the pipe and was not detected on any of the plastic pollution collected at sites A-–F, which may reflect the poor competitive capability of *C. albicans* in the plastisphere.

Adhesion strategies are widely recognised as major virulence factors in pathogenic *Candida*, with several species possessing GPI-modified cell wall proteins (Sundstrom, 2002). The degree of adhesion varies between species, with *C. tropicalis* and *P. kudriavzevii* demonstrating greater adhesion than *C. glabrata* (Modrzewska et al., 2015). Binding to environmental plastic surfaces has previously been demonstrated to increase the virulence of fungal pathogens

(Gkoutselis et al., 2024), but whether biofilm formation on environmental plastic surfaces increases the expression of adhesion factors of environmental isolates of *Candida* and whether this influences subsequent mechanisms of pathogenicity is unknown.

C. glabrata and *C. tropicalis* were the most virulent species isolated from environmental plastic wastes. Invasive candidiasis caused by *C. glabrata* causes 20–50% mortality at 30 days, and *C. tropicalis* responsible for 7% of candidiasis infections, with mortality as high as 60% in adults (WHO, 2022). Whilst *C. albicans* is a common member of the human microbiota, survival on surfaces outside of the human body is limited compared to other *Candida* species (Wißmann et al., 2021). *C. albicans,* which is responsible for 65% of candidiasis infections, was detected upstream of the effluent discharge pipe, but was not isolated from any of the other plastic wastes collected at Sites A–F. Globally, the proportion of *C. albicans* infections is decreasing in parallel with an increasing incidence of *C. glabrata* and *C. tropicalis* (Berkow and Lockhart, 2017), which highlights the importance of quantifying the environmental reservoirs and life-cycle of non-*albicans* species of pathogenic *Candida*.

Human body temperature is a potent nonspecific defence against fungal pathogens, above which opportunistic fungi are usually unable to grow and establish an infection. Several of the environmental isolates of *Candida* in this study were able to grow at 38°C, indicating that they may be capable of overcoming the mammalian thermal defence barrier. After simulating the transfer of *Candida* from the environment to the human body (i.e., initial incubation at 18°C and then moved to 38°C), most environmental isolates of *Candida* showed robust growth. The highest growth rates were demonstrated by

P. kudriavzevii, indicating that it was capable of adapting quickly to a significant temperature change, e.g., by shifting the metabolic pathways from glycerol to trehalose synthesis (Liu et al., 2005). Similarly, *C. glabrata* can tolerate high temperatures due to changes in the calcineurin pathways (Chen et al., 2012). Importantly, several factors that facilitate thermotolerance (e.g., heat-shock protein 90 and calcineurin) can also enhance antifungal resistance (Berman and Krysan, 2020). This is particularly concerning, with rising environmental temperatures as a result of climate change, which could facilitate the development of more resistant and harmful strains of *Candida* (Salazar-Hamm & Torres-Cruz, 2023; Williams et al., 2024).

Less growth was seen when isolates were initially incubated at 38°C before incubation at 18°C (representing the movement of *Candida* from the human body to the environment), indicating lower levels of survival in the environment. However, the protective environment provided by the plastisphere may increase survival of fungi at these temperatures (Lacerda et al., 2020). For all environmental isolates, growth at 28°C was higher, indicating that in warmer locations *Candida* is more likely to survive the transition from the human body to the environment.

C. sojae is an emerging pathogen, and despite its physiological similarities to *C. albicans* and *C. tropicalis,* it was only in 2022 that the first human infection was reported, most likely due to being previously misidentified (Chrenkova et al., 2022). However, in this study, *C. sojae* had lower levels of virulence and antifungal resistance compared to the other species, implying that this species presents a lower risk to public health. There are currently no reports of *C. pseudolambica* causing human infection, although the isolates recovered from

environmental plastics did have high levels of virulence in the *G. mellonella* model. This species is also closely related to pathogenic *P. kudriavzevii* (Ebadi et al., 2022), indicating that there is the evolutionary potential for this species to emerge as a human pathogen. However, *C. pseudolambica* and *C. sojae* showed less growth at 38°C, and both species have lower maximum growth temperatures (below 40°C) compared to other *Candida* species (Ebadi et al., 2022), indicating that temperature may be the main barrier against infection by these two species.

Globally, pathogenic fungi are evolving resistance to all licensed antifungal drugs (Fisher et al., 2022). Azoles, especially fluconazole, are the most widely used antifungals because of their high effectiveness, low toxicity and ability to be taken orally (Partha et al., 2022). However, the environmental isolates screened in this study had high levels of resistance to fluconazole, with several isolates resistant to concentrations of 64 mg/L. Despite *C. albicans* being fairly susceptible to fluconazole, other species of *Candida* such as *C. tropicalis* and *C. glabrata* have relatively high rates of fluconazole resistance. For example, in South Korea fluconazole resistance in *C. glabrata* increased from 0 to 8.3% between 2008 and 2018 (Won et al., 2021), whereas *C. auris* can be highly drug resistant, with 93% of isolates resistant to fluconazole (Lockhart et al, 2017). Although yet to be isolated from environmental plastic pollution, *C. auris* has already been identified in WWTP effluents (Barber et al., 2023) and can persist and remain pathogenic in the plastisphere in simulated environments (Akinbobola et al., 2024).

Voriconazole is an alternative azole, used to treat candidiasis and fluconazole resistant strains of *Candida*; however, there is concern that voriconazole

resistance can emerge following exposure to fluconazole, particularly in cases involving *C. glabrata* (Pfaller et al., 2011). Polyenes and echinocandins are more recently developed antifungals, with higher treatment success rates than azoles (Tashiro et al., 2020). Despite its toxic potential and common side-effects (e.g., nephrotoxicity), amphotericin B is an effective antifungal treatment against progressive and drug-resistant fungal infections with low resistances rates compared to azoles and other antifungals (Cavassin et al., 2021). However, resistance to all anti-fungal drugs is increasing in *Candida*, with evidence of an increase in multi-drug resistance. There are also eco-evolutionary links between environmental and clinical resistance due to the increasing use of agricultural fungicides (particularly azoles), which have been hypothesised to drive increased resistance of *Candida* to antifungal drugs in clinical settings (Fisher et al., 2022).

~~5.~~ Conclusion

Species of *Candida*, including several high priority species on the WHO ‘Fungal priority list’, were isolated from environmental plastic pollution from marine, estuarine, and freshwater ecosystems. Most of these isolates were virulent, thermotolerant, and drug resistant. This highlights a potential public health risk and the urgent need to improve public awareness, monitoring, and environmental management to prevent human exposure to plastic pollution colonised by *Candida*. Climate change and increases in temperature are likely to accelerate the development of resistance and pathogenicity of *Candida*, further highlighting the need for action. Plastics placed downstream of a wastewater effluent pipe were rapidly colonised by several *Candida* species, suggesting a potential point source for human pathogenic *Candida* to be introduced into the environment. With an increase in the prevalence of global infections caused by non-albicans species of *Candida*, it is vital that we increase our focus on these

emerging and recently emerged pathogens and continue to improve our understanding of environmental reservoirs and subsequent transfer routes to humans.

Supplementary information

Table S1. Sampling locations

Site	Site type	Coordinates	Date	Substrate type
A*	Marine	55.640° N, -4.808° W	02/05/23	Sand
B	Estuarine	55.919° N, -4.464° W	02/05/23	Sand/silt
C	Freshwater	55.731° N, -3.906 ° W	02/05/23, 26/05/23	Grass bank, silt riverbed
D	Freshwater	56.021° N, -3.759° W	27/03/23	Mud/silt
E	Marine	55.968° N, -3.136° W	26/05/23	Sand
F*	Marine	55.954° N, -3.109° W	27/03/23	Sand
G	Upstream of WWTP effluent pipe	56.181° N, -3.9631° W	10/05/24	Silt riverbed
H	Downstream of WWTP effluent pipe	56.181° N, -3.9631° W	10/05/24	Silt riverbed

Figure S1. Examples of plastic samples.

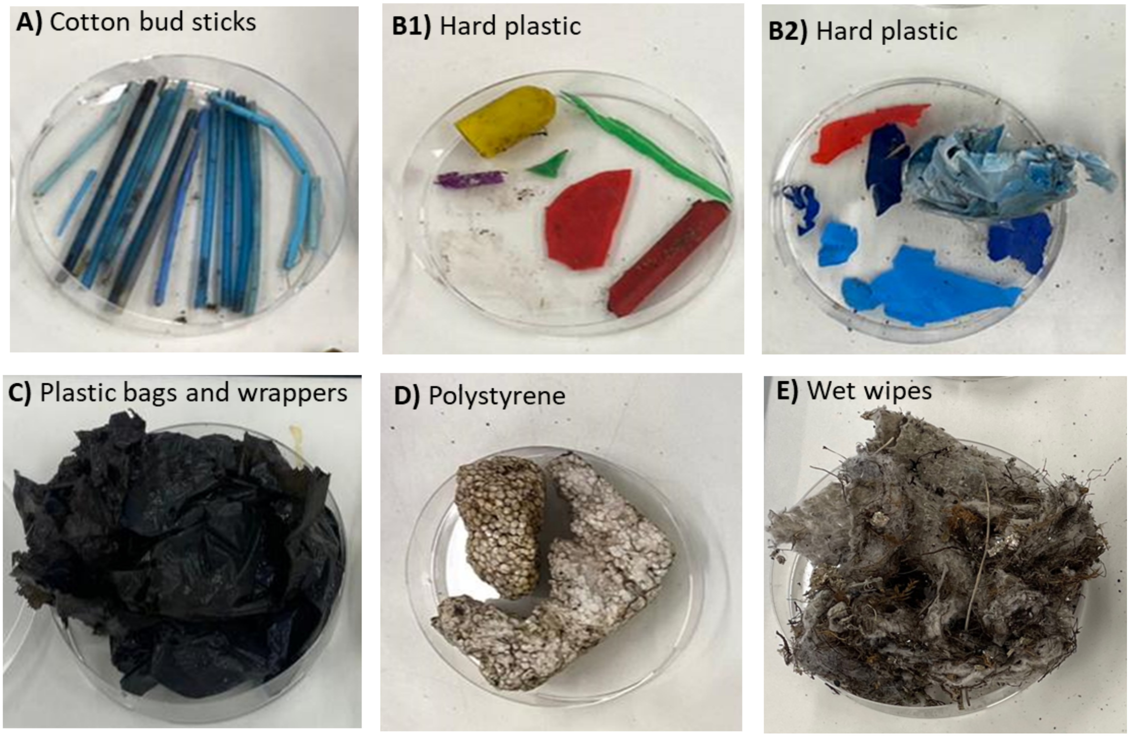

Figure S2. Maximum likelihood phylogenetic analysis of all environmental *Candida* isolates. A 1000 bootstrap iteration tree was generated using a Tamura 3-parameter model and rooted with *Saccharomyces cerevisiae.* The Chromagar Candida plus targets are highlighted as follows; *C. glabrata* (mauve), *P. kudriavzevii* (pink), *C. albicans* (light blue) and *C. tropicalis* (dark blue). Reference sequences for all major targets are shown by their accession numbers.

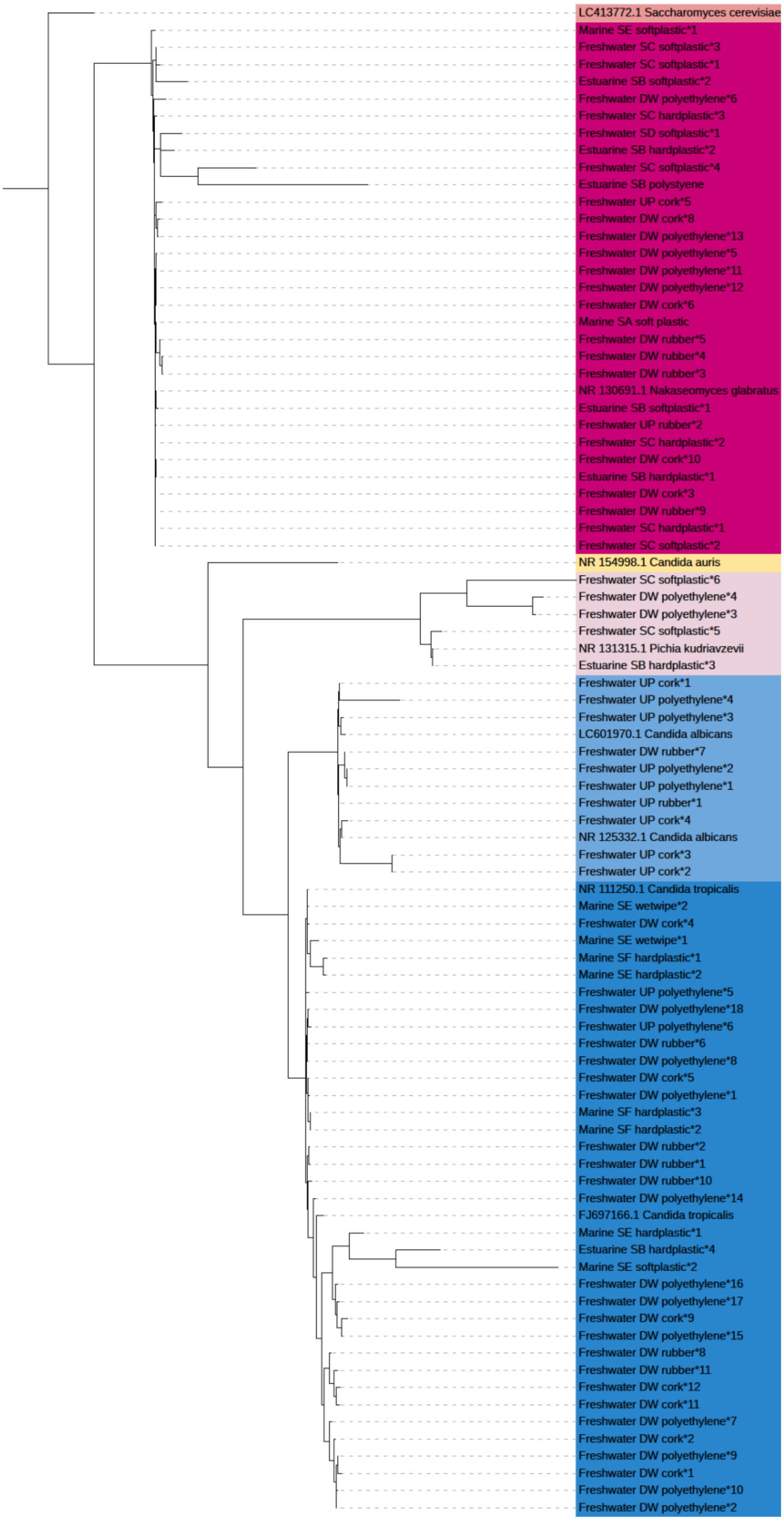

Acknowledgements

None

Author contribution

All authors contributed to the study conception and design. Material preparation and data collection and analysis was performed by Rebecca Metcalf, Ayorinde Akinbobola and Luke Woodford. The first full draft of the manuscript was written by Rebecca Metcalf, and all authors commented on iterative versions of the manuscript. Richard Quilliam provided supervision and funding acquisition. All authors read and approved the final manuscript.

Funding

This work was supported by the UKRI Natural Environment Research Council (NERC) as part of the GCRF SPACES project [grant number NE/V005847/1] and the Plastic Vectors project, "Microbial hitchhikers of marine plastics: the survival, persistence & ecology of microbial communities in the 'Plastisphere'" [grant number NE/S005196/1], both awarded to RQ. The funder had no role in study design, data collection and analysis, decision to publish, or preparation of the manuscript.

Data availability

All datasets are available in the Environmental Information Data Centre repository (EIDCHELP-81892), https://catalogue.ceh.ac.uk/documents/4477f77c-c1c9-44e4-baeb-3353954c8355

Declarations

Ethical approval

Ethical approval for this project was authorised by the General University Ethics Panel (Ref. GUEP 6748) from the University of Stirling.

Consent to participate

No human participants were involved with this study.

Consent for publication

No human participants were involved with this study.

Competing interests

All authors declare they have no competing interests. The funder had no role in study design, data collection and analysis, decision to publish, or preparation of the manuscript.

References

Akinbobola, A.B., Kean, R., Hanifi, S.M.A. and Quilliam, R.S., (2023). Environmental reservoirs of the drug-resistant pathogenic yeast *Candida auris*. PLoS Pathogens, 19(4), p.e1011268.

Akinbobola, A., Kean, R. and Quilliam, R.S. (2024) 'Plastic pollution as a novel reservoir for the environmental survival of the drug-resistant fungal pathogen *Candida auris*.' Marine Pollution Bulletin, 198, 115841.

Arendrup, M.C., Meletiadis, J., Mouton, J.W., Lagrou, K., Hamal, P., Guinea, J., the Subcommittee on Antifungal Susceptibility Testing (AFST) of the ESCMID European Committee for Antimicrobial Susceptibility Testing (EUCAST) (2020) 'EUCAST DEFINITIVE DOCUMENT E.DEF 7.3.2 Method for the determination of broth dilution minimum inhibitory concentrations of antifungal agents for yeasts'.

Arora, P., Singh, P., Wang, Y., Yadav, A., Pawar, K., Singh, A., Padmavati, G., Xu, J. and Chowdhary, A. (2021) 'Environmental isolation of *Candida auris* from the coastal wetlands of Andaman Islands, India'. MBio, 12 (2), pp. 3181.

Assress, H.A., Selvarajan, R., Nyoni, H., Ntushelo, K., Mamba, B.B. and Msagati, T.A., (2019) 'Diversity, co-occurrence and implications of fungal communities in wastewater treatment plants.' *Scientific reports*, *9*(1), p.14056.

Baker, T., Bester, A., Sebolai, O., Albertyn, J. and Pohl, C., 2024. Biofilms on urban aquatic plastic pollution as a reservoir for pathogenic yeasts. *Journal of Water and Health*, *22*(10), pp.1826-1842.

Barber, C., Crank, K., Papp, K., Innes, G.K., Schmitz, B.W., Chavez, J., Rossi, A. and Gerrity, D., (2023) 'Community-scale wastewater surveillance of *Candida auris* during an ongoing outbreak in southern Nevada.' *Environmental Science & Technology*, *57*(4), pp.1755-1763.

Berkow, E.L. and Lockhart, S.R., (2017) 'Fluconazole resistance in *Candida* species: a current perspective.' *Infection and drug resistance*, pp.237-245.

Berman, J. and Krysan, D.J., (2020) 'Drug resistance and tolerance in fungi.' *Nature Reviews Microbiology*, *18*(6), pp.319-331.

Bongomin, F., Gago, S., Oladele, R.O. and Denning, D.W., (2017) 'Global and multi-national prevalence of fungal diseases—estimate precision.' *Journal of fungi*, *3*(4), p.57.

Brandao, L.R., Medeiros, A.O., Duarte, M.C., Barbosa, A.C. and Rosa, C.A., (2010) ‘Diversity and antifungal susceptibility of yeasts isolated by multiple-tube fermentation from three freshwater lakes in Brazil.’ *Journal of Water and Health*, *8*(2), pp.279-289.

Carvalho, A., Costa-De-Oliveira, S., Martins, M.L., Pina-Vaz, C., Rodrigues, A.G., Ludovico, P. and Rodrigues, F., (2007) ‘Multiplex PCR identification of eight clinically relevant *Candida* species.’ Medical Mycology, 45(7), pp.619-627.

Cavassin, F.B., Baú-Carneiro, J.L., Vilas-Boas, R.R. and Queiroz-Telles, F., (2021) ‘Sixty years of amphotericin B: an overview of the main antifungal agent used to treat invasive fungal infections.’ *Infectious Diseases and Therapy*, *10*, pp.115-147.

Chen, Y.L., Konieczka, J.H., Springer, D.J., Bowen, S.E., Zhang, J., Silao, F.G.S., Bungay, A.A.C., Bigol, U.G., Nicolas, M.G., Abraham, S.N. and Thompson, D.A., (2012) ‘Convergent evolution of calcineurin pathway roles in thermotolerance and virulence in *Candida glabrata*.’ *G3: Genes| Genomes| Genetics*, *2*(6), pp.675-691.

Chen, Y.S., Yanagida, F. and Chen, L.Y., (2009) ‘Isolation of marine yeasts from coastal waters of northeastern Taiwan.’ *Aquatic Biology*, *8*(1), pp.55-60.

Chrenkova, V., Vadkertiova, R., Vlachova, K., Babjuk, M., Lischke, R., Bebrova, E. and Hubacek, P., (2022) ‘*Candida sojae*: First report of a human infection.’ Journal de mycologie medicale, 32(4), p.101309.

Denning, D.W., 2024. ‘Global incidence and mortality of severe fungal disease.’ *The Lancet Infectious Diseases*, *24*(7), pp.e428-e438.

Dire, O., Ahmad, A., Duze, S. and Patel, M., 2023. ‘Survival of *Candida auris* on environmental surface materials and low-level resistance to disinfectant.’ *Journal of Hospital Infection*, *137*, pp.17-23.

Ebadi, T., Najafpour, G.D., Younesi, H. and Mohammadi, M., (2022) ‘Rapid biodegradation of diazinon using a novel strain of *Candida pseudolambica*.’ *Environmental Technology & Innovation*, *25*, p.102218.

Ekdahl, L.I., Salcedo, J.A., Dungan, M.M., Mason, D.V., Myagmarsuren, D. and Murphy, H.A., (2023). Selection on plastic adherence leads to hyper-multicellular strains and incidental virulence in the budding yeast. *Elife*, *12*, p.e81056.

Erni-Cassola, G., Zadjelovic, V., Gibson, M.I. and Christie-Oleza, J.A., 2019. Distribution of plastic polymer types in the marine environment; a meta-analysis. *Journal of hazardous materials*, *369*, pp.691-698.

Estivill, D., Arias, A., Torres-Lana, A., Carrillo-Muñoz, A.J. and Arévalo, M.P., 2011. ‘Biofilm formation by five species of *Candida* on three clinical materials.’ *Journal of Microbiological Methods*, *86*(2), pp.238-242.

Fisher, M.C., Alastruey-Izquierdo, A., Berman, J., Bicanic, T., Bignell, E.M., Bowyer, P., Bromley, M., Brüggemann, R., Garber, G. and Cornely, O.A. (2022) ‘Tackling the emerging threat of antifungal resistance to human health.’ Nature Reviews Microbiology, 20 (9), pp. 557-571.

Fisher, M.C., Burnett, F., Chandler, C., Gow, N.A., Gurr, S., Hart, A., Holmes, A., May, R.C., Quinn, J., Soliman, T. and Talbot, N.J., (2024). A one health roadmap towards understanding and mitigating emerging Fungal Antimicrobial Resistance: fAMR. npj Antimicrobials and Resistance, 2(1), p.36.

Fotedar, R., Chatting, M., Kolecka, A., Zeyara, A., Al Malki, A., Kaul, R., Bukhari, S.J., Moaiti, M.A., Febbo, E.J. and Boekhout, T. (2022) ‘Communities of culturable yeasts and yeast-like fungi in oligotrophic hypersaline coastal waters of the Arabian Gulf surrounding Qatar.’ Antonie Van Leeuwenhoek, 115 (5), pp. 609-633.

Geyer, R., Jambeck, J.R. and Law, K.L. (2017) ‘Production, use, and fate of all plastics ever made.’ Science Advances, 3 (7), pp. e1700782.

Gkoutselis, G., Rohrbach, S., Harjes, J., Obst, M., Brachmann, A., Horn, M.A. and Rambold, G. (2021) ‘Microplastics accumulate fungal pathogens in terrestrial ecosystems.’ Scientific Reports, 11 (1), pp. 13214.

Gkoutselis, G., Rohrbach, S., Harjes, J., Brachmann, A., Horn, M.A. and Rambold, G., (2024). Plastiphily is linked to generic virulence traits of important human pathogenic fungi. Communications Earth & Environment, 5(1), p.51.

Gómez-Molero, E., De-la-Pinta, I., Fernández-Pereira, J., Groß, U., Weig, M., Quindós, G., De Groot, P.W. and Bader, O., 2021. Candida parapsilosis colony morphotype forecasts biofilm formation of clinical isolates. *Journal of Fungi*, *7*(1), p.33.

Jiang P, Zhao S, Zhu L, Li D. Microplastic-associated bacterial assemblages in the intertidal zone of the Yangtze Estuary. Science of the Total Environment 2018;624:48–54.

Junaid, M., Siddiqui, J.A., Sadaf, M., Liu, S. and Wang, J., (2022). Enrichment and dissemination of bacterial pathogens by microplastics in the aquatic environment. Science of the total environment, 830, p.154720.

Kainz, K., Bauer, M.A., Madeo, F. and Carmona-Gutierrez, D., (2020) ‘Fungal infections in humans: the silent crisis.’ *Microbial Cell*, *7*(6), p.143.

Kibria, M.G., Masuk, N.I., Safayet, R., Nguyen, H.Q. and Mourshed, M. (2023) ‘Plastic waste: challenges and opportunities to mitigate pollution and effective management.’ International Journal of Environmental Research, 17 (1), pp. 20.

Lacerda, A.L.D.F., Proietti, M.C., Secchi, E.R. and Taylor, J.D., (2020) ‘Diverse groups of fungi are associated with plastics in the surface waters of the Western South Atlantic and the Antarctic Peninsula.’ *Molecular Ecology*, *29*(10), pp.1903-1918.

Lachance, M.A., Boekhout, T., Scorzetti, G., Fell, J.W. and Kurtzman, C.P., (2011). Candida berkhout (1923). In The Yeasts (pp. 987-1278). Elsevier.

Li, W.C., Tse, H.F. and Fok, L. (2016) ‘Plastic waste in the marine environment: a review of sources, occurrence and effects.’ Science of the Total Environment, 566, pp. 333-349.

Liu, H.J., Liu, D.H. and Zhong, J.J., (2005) ‘Interesting physiological response of the osmophilic yeast *Candida krusei* to heat shock.’ *Enzyme and microbial technology*, *36*(4), pp.409-416.

Liu, Y., Liu, W., Yang, X., Wang, J., Lin, H. and Yang, Y., (2021) ‘Microplastics are a hotspot for antibiotic resistance genes: progress and perspective.’ *Science of the Total Environment*, *773*, p.145643.

Lockhart, S.R., Etienne, K.A., Vallabhaneni, S., Farooqi, J., Chowdhary, A., Govender, N.P., Colombo, A.L., Calvo, B., Cuomo, C.A., Desjardins, C.A. and Berkow, E.L., (2017) ‘Simultaneous emergence of multidrug-resistant Candida auris on 3 continents confirmed by whole-genome sequencing and epidemiological analyses.’ *Clinical Infectious Diseases*, *64*(2), pp.134-140.

Maneenil, G., Payne, M.S., Senthamarai Kannan, P., Kallapur, S.G., Kramer, B.W., Newnham, J.P., Miura, Y., Jobe, A.H. and Kemp, M.W., (2015). Fluconazole treatment of intrauterine *Candida albicans* infection in foetal sheep. Pediatric Research, 77(6), pp.740-748.

Mataraci-Kara, E., Ataman, M., Yilmaz, G. and Ozbek-Celik, B., (2020) ‘Evaluation of antifungal and disinfectant-resistant *Candida* species isolated from hospital wastewater.’ *Archives of Microbiology*, *202*, pp.2543-2550.

Medeiros, A.O., Kohler, L.M., Hamdan, J.S., Missagia, B.S., Barbosa, F.A. and Rosa, C.A., (2008) ‘Diversity and antifungal susceptibility of yeasts from tropical freshwater environments in Southeastern Brazil.’ *Water Research*, *42*(14), pp.3921-3929.

Modrzewska, B. and Kurnatowski, P., (2015) ‘Adherence of *Candida* sp. to host tissues and cells as one of its pathogenicity features.’ *Annals of parasitology*, *61*(1).

Morogovsky, A., Handelman, M., Abou Kandil, A., Shadkchan, Y. and Osherov, N., (2022) ‘Horizontal gene transfer of triazole resistance in *Aspergillus fumigatus*.’ *Microbiology Spectrum*, *10*(3), pp.e01112-22.

Oliveira, M.M., Proenca, A.M., Moreira-Silva, E., Dos Santos, F.M., Marconatto, L., de Castro, A.M. and Medina-Silva, R., (2022) ‘Biochemical features and early adhesion of marine *Candida parapsilosis* strains on high-density polyethylene.’ *Journal of Applied Microbiology*, *132*(3), pp.1954-1966.

Parslow, B.Y. and Thornton, C.R. (2022) ‘Continuing shifts in epidemiology and antifungal susceptibility highlight the need for improved disease management of invasive candidiasis.’ Microorganisms, 10 (6), pp. 1208.

Partha, A.D.S.L., Widodo, A.D.W. and Endraswari, P.D., (2022) ‘Evaluation of fluconazole, itraconazole, and voriconazole activity on *Candida albicans*: a case control study.’ *Annals of Medicine and Surgery*, *84*, p.104882.

Pfaller, M.A., Andes, D., Arendrup, M.C., Diekema, D.J., Espinel-Ingroff, A., Alexander, B.D., Brown, S.D., Chaturvedi, V., Fowler, C.L., Ghannoum, M.A. and Johnson, E.M., (2011) ‘Clinical breakpoints for voriconazole and *Candida* spp. revisited: review of microbiologic, molecular, pharmacodynamic, and clinical data as they pertain to the development of species-specific interpretive criteria.’ *Diagnostic microbiology and infectious disease*, *70*(3), pp.330-343.

Plastics Europe, (2022) ‘Annual production of plastics worldwide from 1950 to 2021.’ [Accessed: Retrieved October 09, 2023].

R Core Team. R core team (2021). R: a language and environment for Statistical Computing R Foundation for Statistical Computing, Vienna, Austria URL Http://Www R-Project Org 2021.

Romera, D., Aguilera-Correa, J.J., García-Coca, M., Mahillo-Fernandez, I., Viñuela-Sandoval, L., García-Rodríguez, J., Esteban, J., (2020) 'The *Galleria mellonella* infection model as a system to investigate the virulence of *Candida auris* strains.' Pathogens and disease, 78(9), p.ftaa067.

Salazar-Hamm, P. and Torres-Cruz, T.J., 2024. The impact of climate change on human fungal pathogen distribution and disease incidence. *Current Clinical Microbiology Reports*, *11*(3), pp.140-152.

Sautour, M., Lemaître, J., Ranjard, L., Truntzer, C., Basmaciyan, L., Depret, G., Hartmann, A. and Dalle, F. (2021) 'Detection and survival of *Candida albicans* in soils.' Environmental DNA, 3 (6), pp. 1093-1101.

Seyedmousavi, S., Bosco, S.D.M., De Hoog, S., Ebel, F., Elad, D., Gomes, R.R., Jacobsen, I.D., Jensen, H.E., Martel, A., Mignon, B. and Pasmans, F., (2018). Fungal infections in animals: a patchwork of different situations. Medical mycology, 56(suppl_1), pp.S165-S187.

Silva, S., Negri, M., Henriques, M., Oliveira, R., Williams, D.W. and Azeredo, J., 2011. Adherence and biofilm formation of non-Candida albicans Candida species. *Trends in microbiology*, *19*(5), pp.241-247.

Siscar-Lewin, S., Hube, B. and Brunke, S., (2022) 'Emergence and evolution of virulence in human pathogenic fungi.' *Trends in Microbiology*, *30*(7), pp.693-704.

Sundstrom, P., (2002) 'Adhesion in *Candida* spp.' *Cellular microbiology*, *4*(8), pp.461-469.

Tashiro, S., Osa, S., Igarashi, Y., Watabe, Y., Liu, X., Enoki, Y., Taguchi, K., Mayumi, T., Miyazaki, Y., Takesue, Y. and Matsumoto, K., (2020) 'Echinocandins versus non-echinocandins for the treatment of invasive candidiasis: a meta-analysis of randomized controlled trials.' *Journal of Infection and Chemotherapy*, *26*(11), pp.1164-1176.

Trost, A., Graf, B., Eucker, J., Sezer, O., Possinger, K., Göbel, U.B. and Adam, T., (2004) 'Identification of clinically relevant yeasts by PCR/RFLP.' Journal of microbiological methods, 56(2), pp.201-211.

Wallbank, J.A., Lear, G., Kingsbury, J.M., Weaver, L., Doake, F., Smith, D.A., Audrézet, F., Maday, S.D., Gambarini, V., Donaldson, L. and Theobald, B., (2022) 'Into the Plastisphere, where only the generalists thrive: early insights in plastisphere microbial community succession.' *Frontiers in Marine Science*, *9*, p.841142.

WHO (World Health Organization) (2022) ‘WHO fungal priority pathogens list to guide research, development and public health action’. https://www.who.int/publications/i/item/9789240060241.

Wißmann, J.E., Kirchhoff, L., Brüggemann, Y., Todt, D., Steinmann, J. and Steinmann, E., (2021) ‘Persistence of pathogens on inanimate surfaces: a narrative review.’ *Microorganisms*, *9*(2), p.343.

Wójcik, A., Kurnatowski, P. and Błaszkowska, J. (2013) ‘Potentially pathogenic yeasts from soil of children’s recreational areas in the city of Łódź (Poland).’ International Journal of Occupational Medicine and Environmental Health, 26, pp. 477-487.

Won, E.J., Choi, M.J., Kim, M.N., Yong, D., Lee, W.G., Uh, Y., Kim, T.S., Byeon, S.A., Lee, S.Y., Kim, S.H. and Shin, J.H., (2021) ‘Fluconazole-resistant *Candida glabrata* bloodstream isolates, South Korea, 2008–2018.’ *Emerging infectious diseases*, *27*(3), p.779.

Williams, S.L., Toda, M., Chiller, T., Brunkard, J.M. and Litvintseva, A.P., 2024. Effects of climate change on fungal infections. *PLoS Pathogens*, *20*(5), p.e1012219.

Zettler ER, Mincer TJ, Amaral-Zettler LA. (2013). Life in the “plastisphere”: Microbial communities on plastic marine debris. *Environ Sci Technol* 47, pp. 7137 - 46.

Fig. 1 Sampling sites (black circles) in Central Scotland, UK. Additional site details are given in Table S1.

Table 1 *Candida* isolates from environmental plastic pollution. Species determined by top Genbank accession match and phylogenetic analysis (Fig S2).

‘Hard plastics’, HDPE and PET; ‘soft plastics’ LDPE, PE and PP.

Fig. 2 Virulence of *Candida* isolates colonising environmental plastic pollution (from Sites A–F) in a *Galleria mellonella* infection model. PBS controls are also included (G). Samples are coloured by *Candida* species with the isolate ID shown above. Data points (*n* = ten *G. mellonella* larvae) represent the mean of three independent biological replicates ± SE.

Fig. 3 Thermotolerance of *Candida* isolated from environmental plastic pollution. Isolates were initially incubated for 24 h at 18°C (A–C) or 38°C (D–F), before subsequent transfer to 18°C (A, D), 28°C (B, E) and 38°C (C, F) for a further 24 h. The different coloured bars represent the different species

Fig. 4 Minimum inhibitory concentration (MIC) of four antifungals against *Candida* isolates from environmental plastic pollution. (A) amphotericin B (0.008–4 mg/L); (B) caspofungin (0.008–4 mg/L); (C) fluconazole (0.125–64 mg/L); (D) voriconazole (0.008–4 mg/L). The different coloured bars represent the different *Candida* species. Isolates from sites G and H were only tested against fluconazole at 0.125–32 mg/L. There were three replicates per isolate, and error bars indicate the SE of the mean.
